# Genome-wide characterization and expression profiling of *E2F/DP* gene family members in response to abiotic stress in tomato (*Solanum lycopersicum L*.)

**DOI:** 10.1186/s12870-024-05107-3

**Published:** 2024-05-22

**Authors:** Dhanasekar Divya, Arif Hasan Khan Robin, Lae-Hyeon Cho, Dohyeon Kim, Do-jin Lee, Chang-Kil Kim, Mi-Young Chung

**Affiliations:** 1 Department of Agricultural Education, Sunchon National University, 413 Jungangno, Suncheon, Jeonnam 540-950 Republic of Korea; 2 Department of Genetics and Plant Breeding, Bangladesh Agricultural University, Mymensingh, 2202 Bangladesh; 3 Department of Plant Bioscience, College of Natural Resources and Life Science, Pusan National University, Miryang-si, Gyeongsangnam-do 50463 Republic of Korea; 4 Department of Horticulture, Kyungpook National University, Daegu, 41566 Republic of Korea

**Keywords:** *E2F/DP*, Domain, Tomato, TF, Abiotic stress, miRNA, Proteins

## Abstract

**Background:**

E2F/DP (Eukaryotic 2 transcription factor/dimerization partner) family proteins play an essential function in the cell cycle development of higher organisms. E2F/DP family genes have been reported only in a few plant species. However, comprehensive genome-wide characterization analysis of the E2F/DP gene family of *Solanum lycopersicum* has not been reported so far.

**Results:**

This study identified eight nonredundant *SlE2F/DP* genes that were classified into seven groups in the phylogenetic analysis. All eight genes had a single E2F-TDP domain and few genes had additional domains. Two segmental duplication gene pairs were observed within tomato, in addition to cis-regulatory elements, miRNA target sites and phosphorylation sites which play an important role in plant development and stress response in tomato. To explore the three-dimensional (3D) models and gene ontology (GO) annotations of SlE2F/DP proteins, we pointed to their putative transporter activity and their interaction with several putative ligands. The localization of SlE2F/DP-GFP fused proteins in the nucleus and endoplasmic reticulum suggested that they may act in other biological functions. Expression studies revealed the differential expression pattern of most of the *SlE2F/DP* genes in various organs. Moreover, the expression of *E2F/DP* genes against abiotic stress, particularly *SlE2F/DP*2 and/or *SlE2F/DP*7, was upregulated in response to heat, salt, cold and ABA treatment. Furthermore, the co-expression analysis of *SlE2F/DP* genes with multiple metabolic pathways was co-expressed with defence genes, transcription factors and so on, suggested their crucial role in various biological processes.

**Conclusions:**

Overall, our findings provide a way to understand the structure and function of *SlE2F/DP* genes; it might be helpful to improve fruit development and tolerance against abiotic stress through marker-assisted selection or transgenic approaches.

**Supplementary Information:**

The online version contains supplementary material available at 10.1186/s12870-024-05107-3.

## Background

Due to various biotic and abiotic stresses, plants have evolved complex signalling pathways for their defence mechanism [1]. The innate mechanism in plants induces the release of secondary metabolism and phytohormones, signalling receptors and signal transducers to active defence pathways [2]. Some signalling pathways are reported to be common in plants against both abiotic and biotic stress [3]. Different abiotic stresses, such as heat, cold, drought, and salt, trigger plant sensors by a single or simultaneous attack to activate the resistance mechanism [4, 5]. Many of the transcription factor (TFs) regulate gene expression by binding to cis-acting elements in the promoter region against abiotic stress [6, 7]. Transcription factors (TF) induce the genes involved in cell signalling, plant hormone metabolism, pathogenesis-related protein and in primary metabolism and secondary metabolism of defence (SAR). Till now, around 320k TFs have been reported from 165 different plant species. The most-known TF families are WRKY, MBS, MADS, ARE, bZIP, GRAS etc [7–9]. In addition, some sub-category TF families, SNF7, CPP, and E2F/DP are also reported to be involved in defence against heat stress in *Arabidopsis* and rice [10].

In plants and animals, the Eukaryotic 2 transcription factor/dimerization partner transcription factor family (henceforth E2F/DP) is reportedly involved in the cell cycle and its development. The *E2F/DP* transcription factors in plants are categorized into E2F, DP, and DEL (DP-E2F-like) groups based on their conserved domains. The E2F gene group contains four functional domains: an RBR-binding domain, a DNA-binding domain, a ‘marked box’ domain and a leucine zipper dimerization domain [11]. However, the members of the DP group contain a DNA-binding domain and/or leucine zipper dimerization domain when compared with the E2F genes. The DEL group of genes has only a DNA-binding domain [11].

The *E2F/DP* TFs are reportedly involved in cell growth and proliferation in mammalian E2F signalling pathways [12]. Likewise, in *Arabidopsis* plants the E2F signalling pathway functions as a core regulator with cyclin-dependent kinase inhibitors (CKIs), retinoblastoma (RBs), cyclins, and cyclin-dependent kinases (CDKs). In plants and mammals, the *E2F* transcription factor binds to the TTTCCCGCC motif with or without slight modification in its promoter region of the target genes [13]. Another study of E2F family gene structural analysis revealed that most of the genes shared similar intron numbers/lengths within the same groups.

In *Arabidopsis*, the *E2Fa* and *E2Fb* genes bind with DP to regulate their gene expression [14]. Similarly, in Moso bamboo, the *PheE2F/DP* genes were reportedly involved in leaf and root growth, genome integrity and viability, and abiotic stress response [14–18]. Most of the *PheE2F/DP* genes were upregulated against drought and salt stress at different time points. Similarly, four genes (*E2Fa, E2Fb*, *E2Fc* and *E2FDPa*) showed more than 2-fold against salt stress in *Medicago truncatula* [18, 19]. In wheat (*Triticum aestivum*), *TaE2F1I-19* and *TaDP3III-15* were upregulated against drought stress; *TaDP2III-3* and *TaDEL2II-27* genes were expressed against salt stress and heat stress; *TaE2F1I-19* and *TaDP3III-15* were significantly upregulated against cold stress [20].

Tomato (*Solanum lycopersicum*) is a model vegetable crop cultivated worldwide, and it stands in the fourth position among the leading vegetables in terms of production [21]. Tomato is grown on an area of 5.06 million hectares worldwide with an annual production of 182.05 million tons [22]. Biotic and abiotic stress affects a significant amount of tomato production, which directly affects fruit development and ripening [23]. Some of the major biotic stresses reported in tomatoes are bacterial wilt and spot, cucumber mosaic virus, cotton bollworm, nematodes and so on [21]. Similarly, some of the reported abiotic stresses in tomatoes are temperature, light, salt, cold, and drought [24]. It has been estimated that around 70% of yield losses yearly are due to abiotic and biotic stress in tomato [24]. It is a rich source of nutrients such as lycopene, lutein, zeaxanthin, potassium, ascorbic acid, and β-carotene [25]. Advanced biotechnological tools have been introduced to new dimensional omics disciplines such as transcriptomics, proteomics and metabolic studies. Several genome-wide characterizations of genes were reported after the release of the tomato genome sequence in 2012 [26].

The genome-wide identification and characterization of gene families will provide the following parameters: chromosomal distribution, number of introns and exons, phylogenetic relationship, gene duplication, expression profiles and so forth that can be used for tomato crop improvement [27]. A genome-wide characterization of the E2F/DP gene family has been performed in a few plant species. Nevertheless, no such efficient investigation of the evolutionary relationships or characteristics of the E2F/DP family genes has been performed in solanaceous crops. Therefore, in the present study, we studied the gene expression of *SlE2F/DP* genes in different organs and against five abiotic stresses at different time points in tomato. In addition to the gene structure, cis-acting elements, miRNA prediction in SlE2F/DP proteins, subcellular localization assays and gene co-expression network analysis using RNA sequencing data were also carried out to understand their probable functional role in tomato. Our findings aimed to provide a basis for further functional verification of potential *E2F/DP* transcription factors in tomatoes for crop improvement programs.

## Results

### *In silico* identification of*E2F/DP* in tomato

This study identified eight *E2F/DP* tomato genes and named them Sl*E2F/DP*1–Sl*E2F/DP*8 based on their chromosomal locations. The ORFs of *SlE2F/DP* showed significant variation in length, ranging from 588 bp (*SlE2F/DP*6) to 2554 bp (*SlE2F/DP*5), with a mean of 1309 bp. Similarly, the lengths of the E2F/DP proteins varied from 195 to 847 amino acids (aa) for SlE2F/DP6 and SlE2F/DP5, respectively, with a mean of 435 aa. In addition, the predicted molecular weights (MW) vary from 22.2 to 93.2 kDa. The isoelectric point (pI) values of SlE2F/DP proteins varied between 4.68 and 8.92, indicating that E2F/DPs may be acidic or basic. The GRAVY values ranged from − 0.505 to − 0.734, for SlE2F/DP proteins indicating that all these proteins are hydrophilic in nature. Additional information such as subcellular location, chromosome location, and ORF, of these SlE2F/DP family members is given in Table 1.

**Table 1:** List of identified tomato *E2F/DP* gene family members with their corresponding encoded protein information

Gene name	Locus name	ORF (bp)	Chromosome No	Length (aa)	Domain start- end (aa)	MW (kDa)	PI	GRAVY	Subcellular localization	No. of introns
*SlE2F/DP1*	Solyc01g007760.2.1	1440	1	479	237–337	52.58	4.97	-0.634	Chloroplast	13
*SlE2F/DP2*	Solyc02g087310.2.1	1125	2	374	21–86	42.15	8.84	-0.707	Nucleus	10
*SlE2F/DP3*	Solyc03g113760.2.1	1158	3	385	18–82	43.27	8.92	-0.595	Nucleus	10
*SlE2F/DP4*	Solyc04g081350.2.1	1194	4	397	213–319	44	5.79	-0.563	Nucleus	12
*SlE2F/DP5*	Solyc06g074010.2.1	2554	6	847	200–306	93.15	4.72	-0.552	Nucleus	27
*SlE2F/DP6*	Solyc09g010670.2.1	588	9	195	76–180	22.22	5.14	-0.505	Nucleus	6
*SlE2F/DP7*	Solyc10g078430.1.1	1065	10	354	188–326	38.42	6.28	-0.734	Nucleus	8
*SlE2F/DP8*	Solyc11g068800.1.1	1350	11	449	209–309	49.69	4.68	-0.635	Nucleus	13

### Phylogenetic analysis of tomato E2F/DP family proteins

**Fig. 1:**
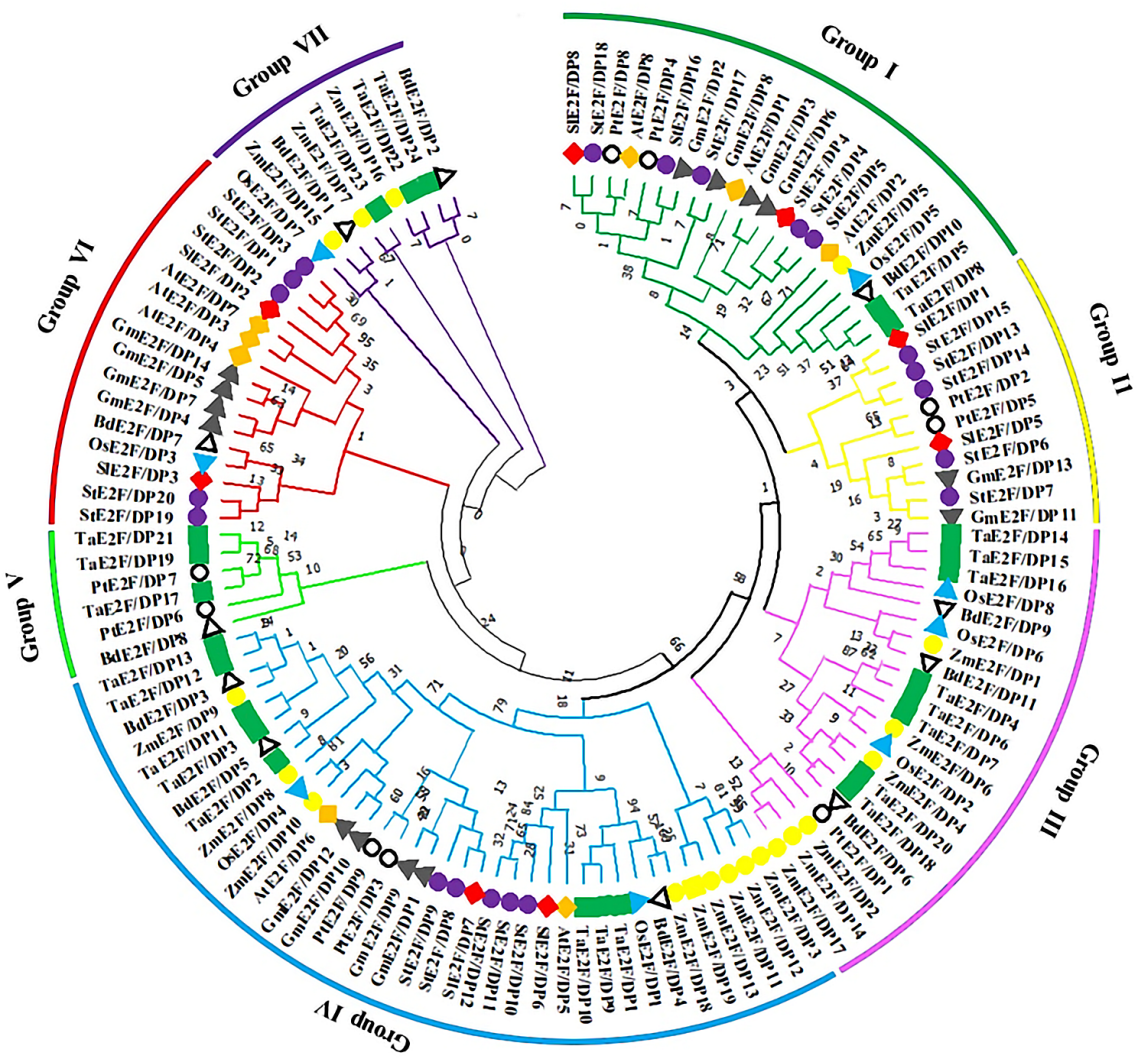
Phylogenetic analysis of E2F/DP proteins from tomato and different plant species. The neighbor-joining tree was constructed with the full length E2F/DP proteins using ClustalW and MEGA11 with 1000 bootstrap replicates. A species abbreviation was provided prior to each E2F/DP protein name: Sl, *Solanum lycopersicum*; St, *Solanum tuberosum*; At, *Arabidopsis thaliana*; Gm, *Glycine max*; Os, *Oryza sativa*; Bd, *Brachypodium distachyon*; Zm, *Zea mays*; Pt, *Populus trichocarpa* and Ta, *Triticum aestivum*

The protein sequence homology ranged from 9 to 71% among SlE2F/DP proteins (Table S1), and those proteins that were clustered in the same phylogenetic groups had higher sequence similarity among the nine different plant species (Table S13). The phylogenetic analysis classified diverse plant E2F/DP family proteins into seven different groups based on their phylogenetic association (Fig. 1). Mainly, all the proteins of monocots (stiff brome, maize, rice, and wheat) and dicots (black cottonwood, tomato, soybean, *Arabidopsis*, and potato) split into two different groups in the phylogenetic analysis. The eight tomato E2F/DPs were distributed in four different groups, with a similar number of proteins (SlE2F/DP8 and SlE2F/DP4) in group I and another two proteins (SlE2F/DP1 and SlE2F/DP5) in group III. The other two groups had two proteins each: group IV with SlE2F/DP6 and SlE2F/DP7, and group VI confined with SlE2F/DP2 and SlE2F/DP3 proteins. All SlE2F/DP protein members preferentially clustered with their homologs from potato, which is an evolutionarily closely related species. All the dicotyledonous species were clustered into groups 1, 2 and 6, and monocot homologs were clustered into groups 3, 5 and 7. In contrast, group four clustered both monocot and dicot *E2F/DP* homologs in the evolutionary relationship.

### Gene structure, conserved motif and domain analysis of tomato *E2F/DP* genes

**Fig. 2:**
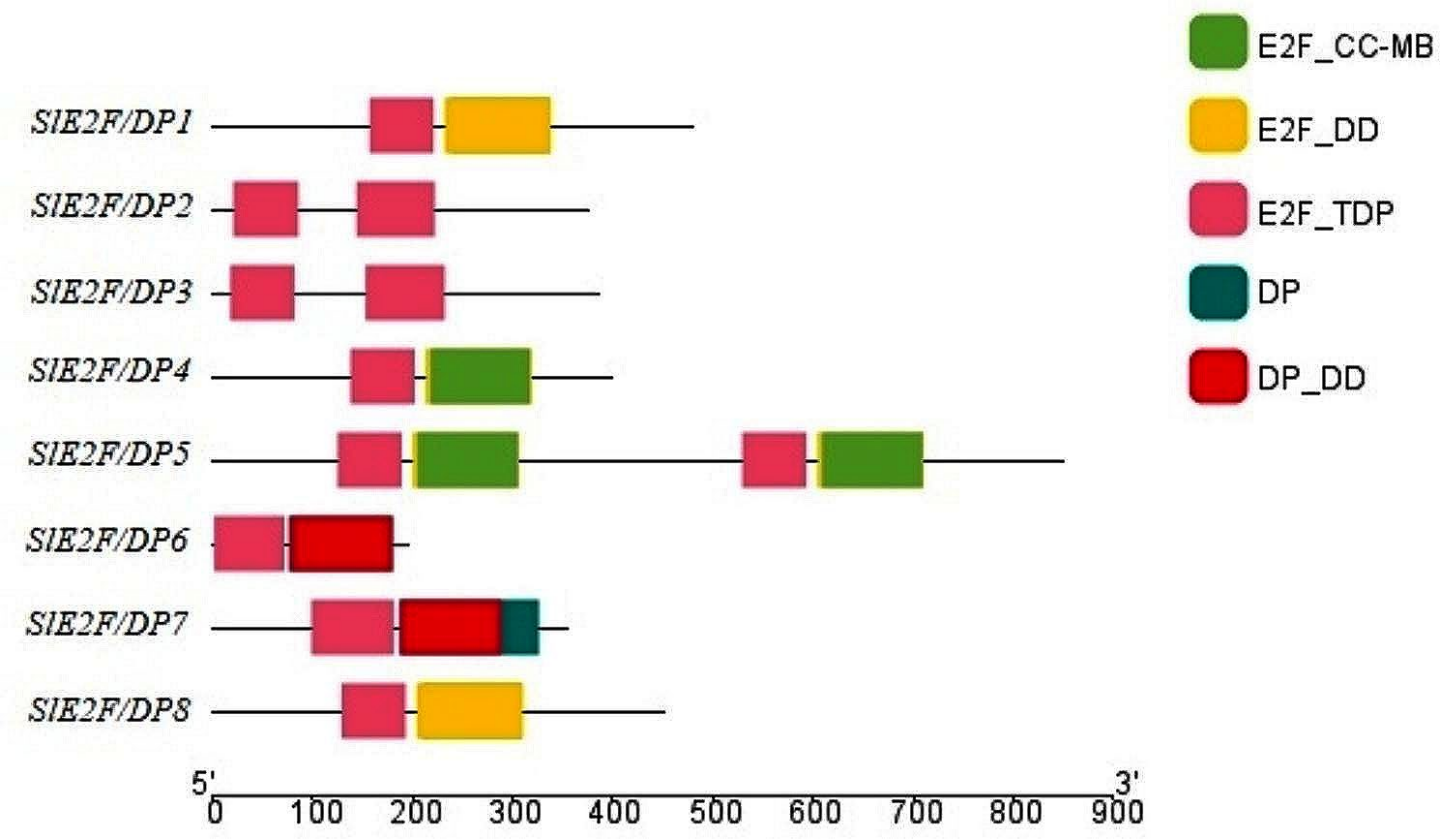
Schematic depiction of the domain organization of SlE2F/DP proteins. E2F_TDP domain, E2F_CC-MB domain, E2F_DD domains, DP domains and DP_DD superfamily domain identified in SlE2F/DP proteins are shown

**Fig. 3:**
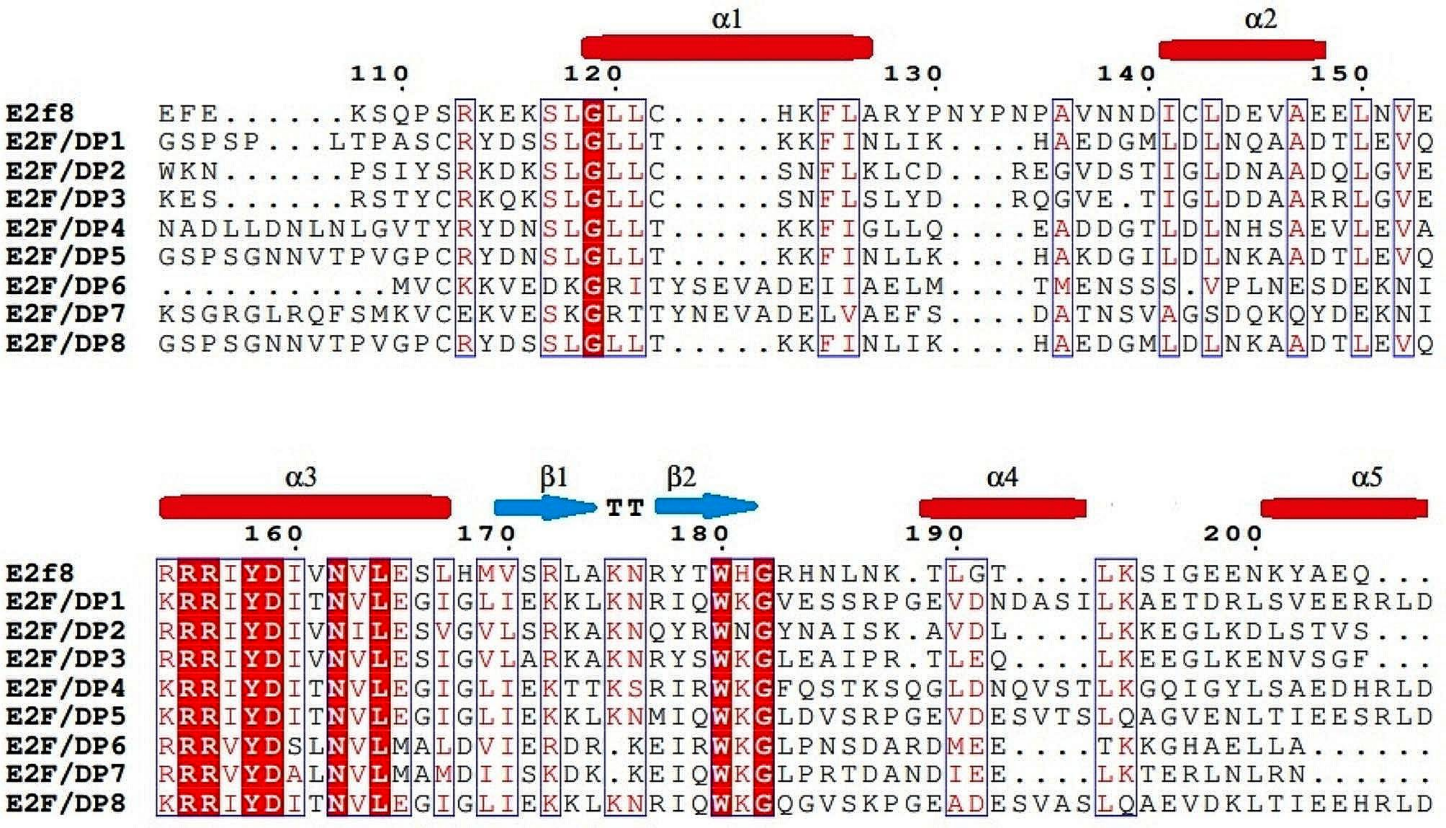
Alignment of E2F/DP domains of SlE2F/DP proteins with that of the typical human E2F8. The secondary structural elements determined by the Espript 3.0 web tool are indicated above the alignment

Exon-intron analysis revealed structural divergence in the tomato E2F/DP gene family (Fig. S1). The number of exons present in the *SlE2F/DP* genes varied from seven to twenty-eight, with a mean of thirteen. However, the exon-intron composition of the *E2F/DP* genes within the same phylogenetic group was similar. Likewise, most of the *SlE2F/DP* homologs clustered in the same phylogenetic groups contained similar conserved motif distributions, (Fig. S2, Fig. S3). This study also identified several motifs unique to some *E2F/DP* members, such as motif 5, motif 8 and motif 9 for *SlE2F/DP*2 and *SlE2F/DP*3, motif 4 for *SlE2F/DP*6 and *SlE2F/DP*7, and motif 2 and motif 10 for *SlE2F/DP*1, *SlE2F/DP*4, *SlE2F/DP*5 and *SlE2F/DP*8 genes. The conserved motifs predicted among the homologous proteins from three different species (*Arabidopsis*, tomato, and rice) were clustered in the same phylogenetic groups. Motifs 1 and 6 were present in all E2F/DP homologous proteins of *Arabidopsis*, tomato, and rice, indicating that these motifs were conserved in both dicot and monocot plants during evolution. The domain architecture of *SlE2F/DP* genes were assorted, changing from the presence of the single E2F_TDP domain to the composition of other additional domains, such as the E2F_CC-MB domain, E2F_DD domains, DP domains and DP_DD superfamily domain (Fig. 2). The E2F_CC-MB domains were identified only in the *SlE2F/DP*4 and *SlE2F/DP*5 genes. Similarly, another domain, E2F_DD, was identified only in the *SlE2F/DP*1 and *SlE2F/DP*8 genes. Other types of domains, DP and DP_DD, were only identified in the *SlE2F/DP*6 and *SlE2F/DP*7 genes. Sequence alignments of SlE2F/DP family members with the human homolog E2F8 protein revealed that the E2F/DP family proteins from a human and a dicotyledonous tomato plant shared five α-helices, β-turns labeled as TT and two β-sheets (Fig. 3).

### Chromosomal position, gene duplication, and microsynteny analysis of *SlE2F/DP* genes

**Fig. 4:**
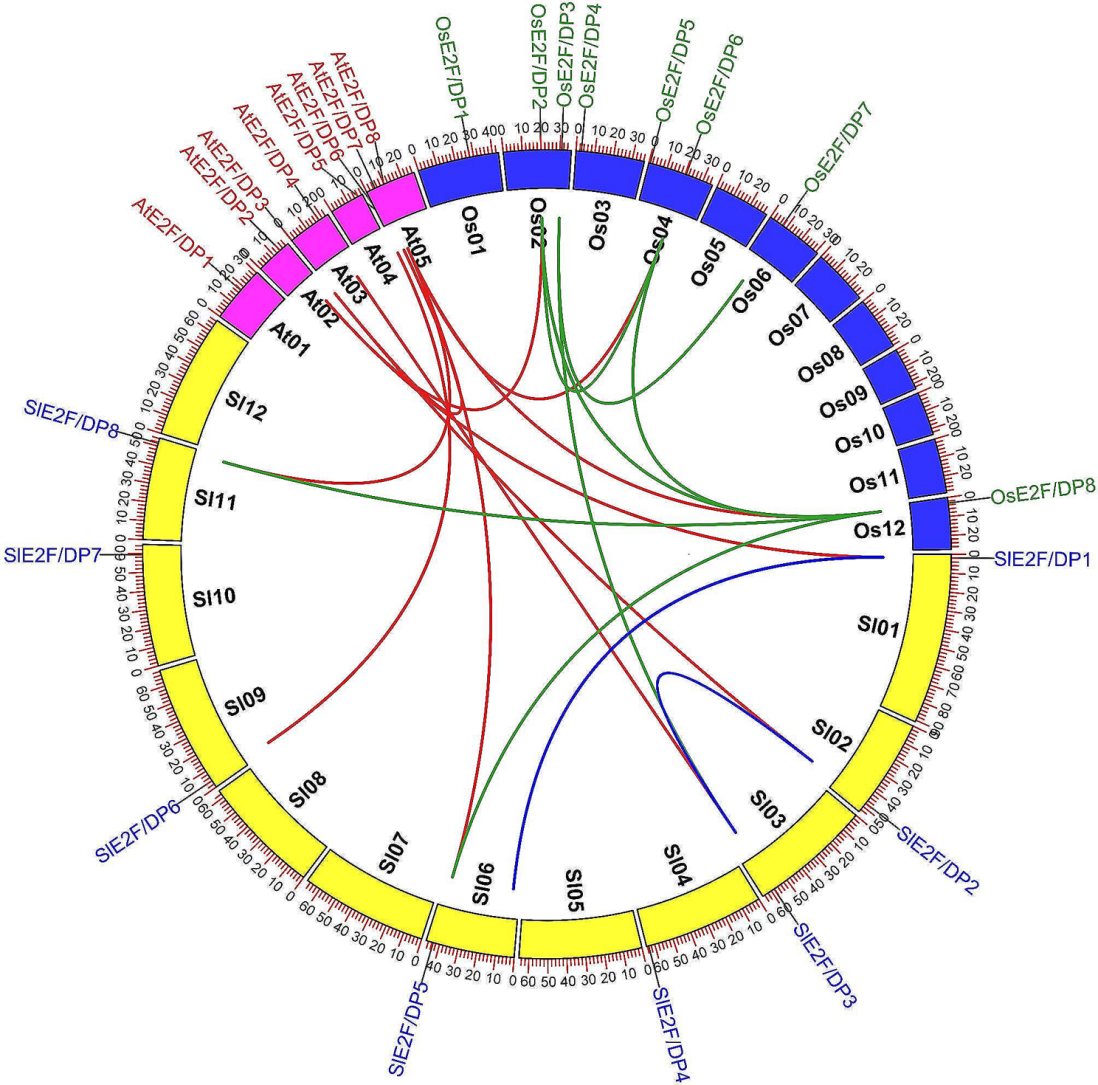
Microsyntenic relationship of *E2F/DP* genes across *Arabidopsis*, tomato, and rice. The chromosomes of the three species are depicted by different colors: *Arabidopsis*, pink; tomato, yellow; and rice, blue. All chromosomes are showed with the scale in megabase pairs (Mbp). The duplicated *SlE2F/DP* genes in tomato genome are indicated by blue lines,

*SlE2F/DP* genes were evenly distributed on 8 of the 12 chromosomes (Chr) of tomato, and they were placed near the distal portions of the chromosomes (Fig. S4). Two segmentally duplicated gene pairs (*SlE2F/DP*1 and *SlE2F/DP*5 and *SlE2F/DP*2 and *SlE2F/DP*3) were predicted within the tomato genome, one duplicate pair clustered into phylogenetic group VI, whereas other pairs distributed in group II and group I (Fig. 1, Fig. S5). However, all the duplicated genes of each pair were located on different chromosomes with a single *SlE2F/DP* gene. This study did not detect tandem duplication events because all the genes were mapped on different chromosomes. The Ka/Ks ratio was less than one for the two duplicated gene pairs in the tomato E2F/DP gene family suggested that these genes duplicated due to intense purifying selection over the course of evolution. Similarly, the Ka/Ks ratio was less than one for the duplicated gene pairs between tomato and *Arabidopsis* (Table 2). It has been estimated that around 13.15–14.76 million years ago these duplicated gene pairs separated. The time estimation was ranged between 49.51 and 142.41 million years ago for the duplicated gene pairs of *Arabidopsis* and tomato. The comparative microsynteny analysis was also conducted among *Arabidopsis*, tomato, and rice *E2F/DP* orthologous genes to distinguish the evolutionary correlation across the genomes (Fig. 4). This analysis identified six orthologous gene pairs between *Arabidopsis* and tomato, whereas three pairs between tomato and rice and three pairs between *Arabidopsis* and rice.

**Table 2:** Predicted Ka/Ks ratio of the duplicated *SlE2F/DP* and *AtE2F/DP* gene pair along with its divergence time

No	Paralogous pair	Ka	ks	ka/ks	Duplication	Time (MYA)
1	*SlE2F/DP1* and *SlE2F/DP5*	0.157	0.3946	0.354682	Segmental	13.15
2	*SlE2F/DP2* and *SlE2F/DP3*	0.185	0.4427	0.468576	Segmental	14.76
3	*AtE2F/DP1 and SlE2F/DP1*	0.3320	2.5436	0.130556	Segmental	84.78
4	*AtE2F/DP2 and SlE2F/DP2*	0.4497	1.4855	0.30273	Segmental	49.51
5	*AtE2F/DP2 and SlE2F/DP2*	0.4088	2.8606	0.142926	Segmental	95.51
6	*AtE2F/DP2 and SlE2F/DP2*	0.2835	3.4194	0.082934	Segmental	113.98
7	*AtE2F/DP2 and SlE2F/DP2*	0.3368	4.2723	0.078835	Segmental	142.41
8	*AtE2F/DP2 and SlE2F/DP2*	0.2862	1.9240	0.148769	Segmental	64.13

Ks, the number of synonymous substitutions per synonymous site; Ka, the number of non-synonymous substitutions per nonsynonymous site; MYA, Million Years Ago

### Prediction of *Cis*-regulatory elements, microrna (mirna) target sites and phosphorylation sites

Hormonal regulation is essential for activating gene expression to control plant stress responses [28]. This study identified many *cis*-regulatory elements related to phytohormones and abiotic stress responses in the promoter regions of tomato *E2F/DP* genes. They are drought‐responsive MYB‐binding site (MBS) and MYB elements, stress/defense‐related elements (TC‐rich repeats), jasmonic acid‐responsive elements (CGTCA‐motif), related to the SA response (TCA‐elements), auxin responsive elements (TGA‐element and TGA‐box), ABA‐responsive elements (ABRE), low‐temperature‐responsive elements (LTR), and GA responsive elements (P‐box, GARE‐ and TATC) (Fig. S6, Table S2). In addition to cis-acting elements, miRNAs also regulate stress-related genes against biotic and abiotic stress in plants [29, 30]. This study identified six miRNA target sites related to abiotic stress tolerance in tomato in *SlE2F/DP*1, *SlE2F/DP*2, *SlE2F/DP*4 and *SlE2F/DP*8 genes. The remaining four genes were not predicted to have miRNA target sites (Table S3). Post‐translational regulation of stress‐related proteins via phosphorylation is important in plant stress responses. This study identified several phosphorylation sites, including CKI and CKII, in tomato SlE2F/DP proteins (Table S4).

### Comparative modeling of tomato E2F/DP proteins

**Fig. 5:**
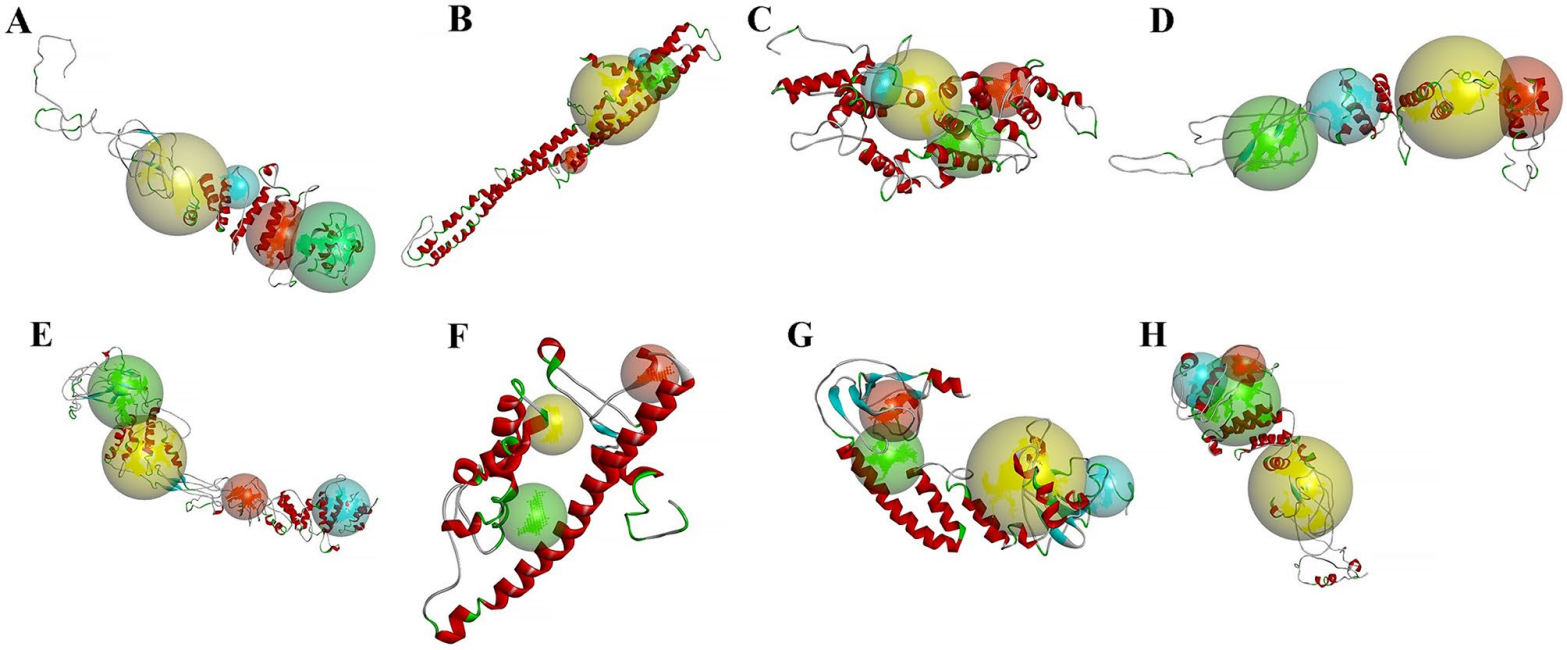
Predicted three-dimensional homology structure of tomato E2F/DP proteins. The final 3D structures of SlE2F/DP proteins were built by Discovery Studio v.21.1. The secondary structural components: α‐helices (red), β‐sheets (cyan), coils (green), and loops (gray) as well as the top four putative binding sites: site 1 (yellow sphere), site 2 (green sphere), site 3 (red sphere), and site 4 (blue sphere) are indicated in the predicted 3D models of (**A**) SlE2F/DP1; (**B**) SlE2F/DP2; (**C**) SlE2F/DP3; (**D**) SlE2F/DP4; (**E**) SlE2F/DP5; (**F**) SlE2F/DP6; (**G**) SlE2F/DP7 and (**H**) SlE2F/DP8

All these SlE2F/DP proteins were modeled for the 3D protein structures using the online server I-TASSER along with the C-score, sequence similarity percent, coverage and binding sites (Tables S5 and S6). Using the binding sites, rotating 3D models were created using the software Discovery Studio v.21.1. The number of secondary structural components of tomato E2F/DP proteins was 5–17 for α-helixes, 6–14 for β‐strands, and 13–29 for coils (Table S5). However, the number of secondary structures differed, one protein had few α‐helixes, β‐strands and coils (5, 7 and 13 for SlE2F/DP6), whereas, other proteins had high α‐helixes and coils (17 and 29 for SlE2F/DP2). Along with the secondary structure, the C‐scores and TM‐scores were predicted for all the protein models in Tables S6 and Table S7 [31]. This study identified the putative ligand‐binding sites that can interact with diverse molecules in all predicted models (Fig. 5). To understand the molecular functions of SlE2F/DP family members, gene ontology (GO) terms were used to determine the binding ability to a variety of ligands, transporters and transferase activity using the online server I‐TASSER (Table S8).

### Co-expression analysis

**Fig. 6:**
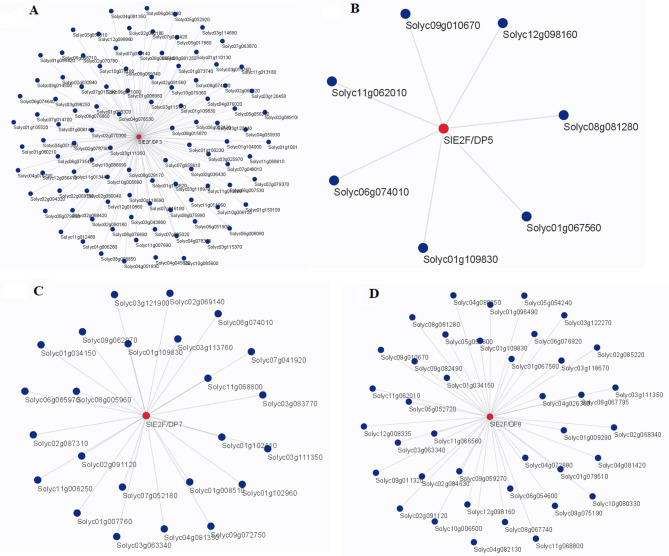
Weighted gene co-expression network analysis (WGCNA) of *SlE2F/DP* genes. A–D: the co‐expressed genes in the network of (**A**) *SlE2F/DP3*, (**B**) *SlE2F/DP5*, (**C**) *SlE2F/DP7* and (**D**) *SlE2F/DP8*. The *SlE2F/DP* genes are marked in red

Co-expression profiling was performed for the *SlE2F/DP* genes using RNA sequencing data. The results showed that a total of 166 genes were co‐expressed with *SlE2F/DP* genes (Fig. 6). The hub genes, *SlE2F/DP*3, *SlE2F/DP*5, *SlE2F/DP*7, and *SlE2F/DP*8 were co‐expressed with 102, 7, 24, and 33 genes, respectively. The KEGG enrichment analysis of these co-expressed genes was concerned with biological pathways, including nucleotide excision repair, pyrimidine metabolism, base excision repair, amino sugar and nucleotide sugar metabolism, basal transcription factors, biosynthesis of secondary metabolites, one carbon pool by folate, phenylpropanoid biosynthesis and Folate biosynthesis (Fig. S7, Fig. S8, Table S9). However, some of the co-expressed genes were not annotated in any of these pathways. In this study, a few of the defence response genes namely *HSP* (*Solyc01g100230*), *NBS-LRR* (*Solyc08g005510*), *F-box-LRR* (*Solyc10g085600*), *NAC domain* (*Solyc02g036430*), *WD40 domain* (*Solyc03g114690*), and *B3 domain* (*Solyc06g007530*) were co-expressed with the *SlE2F/DP3* gene. Furthermore, the *SlE2F/DP*5 gene was co-expressed with a set of genes such as *thymidylate synthase* (*Solyc01g109830*), which is associated with tomato fruit ripening and *AEP* (*Solyc12g098160*). Similarly, another *HSP20* gene (*Solyc01g102960*) and *GDSL* (*Solyc11g006250*) gene involved in plant growth and development were co-expressed with the *SlE2F/DP7* gene. Likewise, a few transcription factors namely *zinc finger C3HC4* and *CCCH domain* (*Solyc05g051800* and *Solyc06g054600*), *serine/threonine* gene (*Solyc09g011320*) reported against biotic and abiotic stress, *cyclin related* gene (*Solyc04g072880*) involved in the signalling control of stress tolerance were co-expressed with the hub gene *SlE2F/DP*8.

### Subcellular localization of SlE2F/DP proteins

**Fig. 7:**
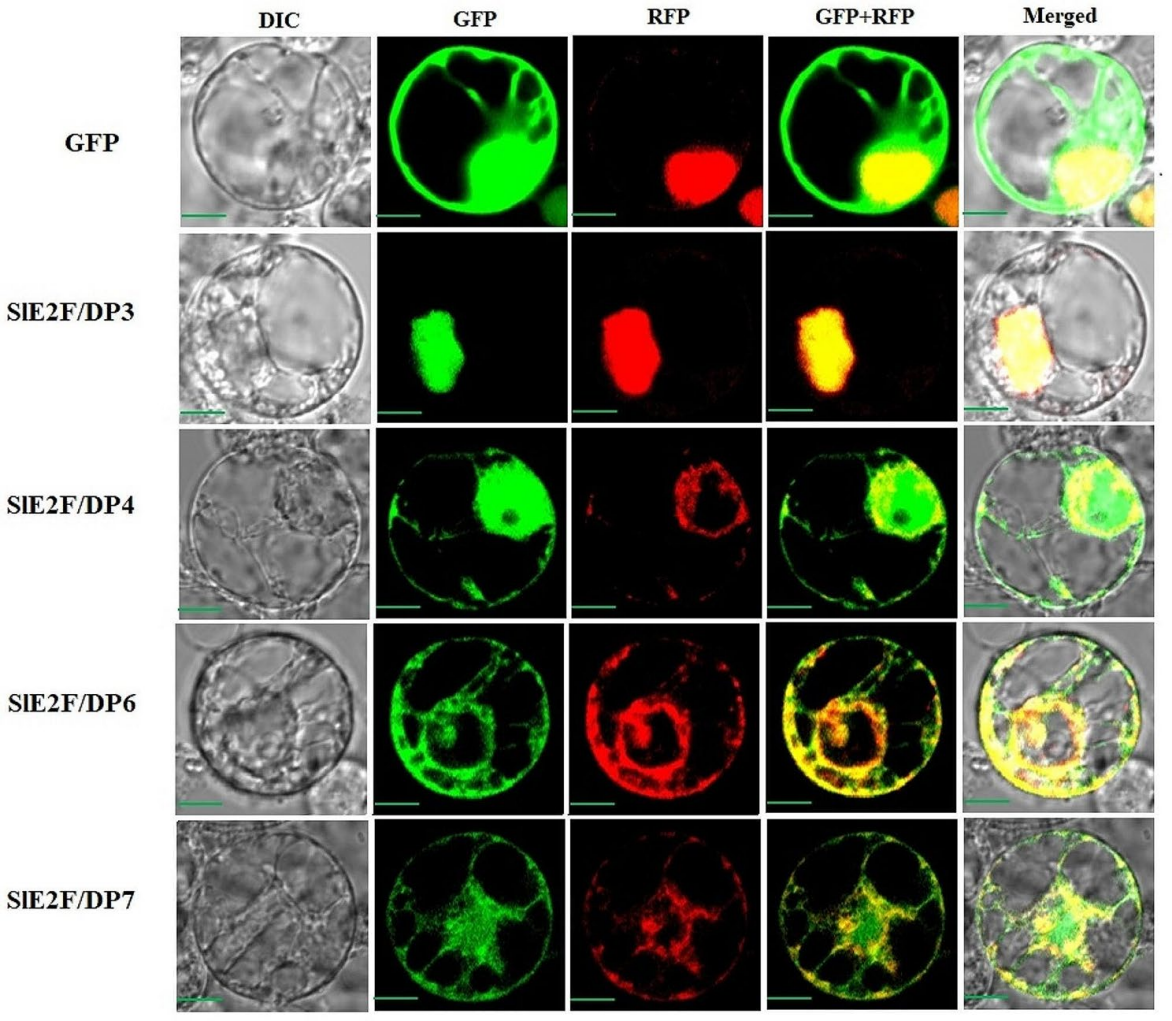
Subcellular localization of SlE2F/DP proteins. SlE2F/DPs-SGFP fusion constructs were used to analyze the localization of SlE2F/DP3, SlE2F/DP4, SlE2F/DP6 and SlE2F/DP7 and the fluorescence signals were visualized with the confocal microscope. The NLS-mRFP construct and ER-mRFP was utilized as an Nucleus and ER localization marker. Scale bars = 10 μm

The SlE2F/DP family genes were predicted for localization analysis using the wolfsport server. The E2F/DP proteins were predicted in various parts of the cell, such as the nucleus, ER, and cytoplasm (Table S10). Furthermore, to validate the *insilico* predicted results, subcellular localization was carried out by expressing these SlE2F/DP proteins fused with sGFP in rice protoplasts. The results showed that SlE2F/DP4, SlE2F/DP6 and SlE2F/DP7 had florescence signal in the ER whereas, SlE2F/DP3 was localized in the nucleus (Fig. 7).

### Expression profiling of tomato*E2F/DP*genes in different organs

**Fig. 8:**
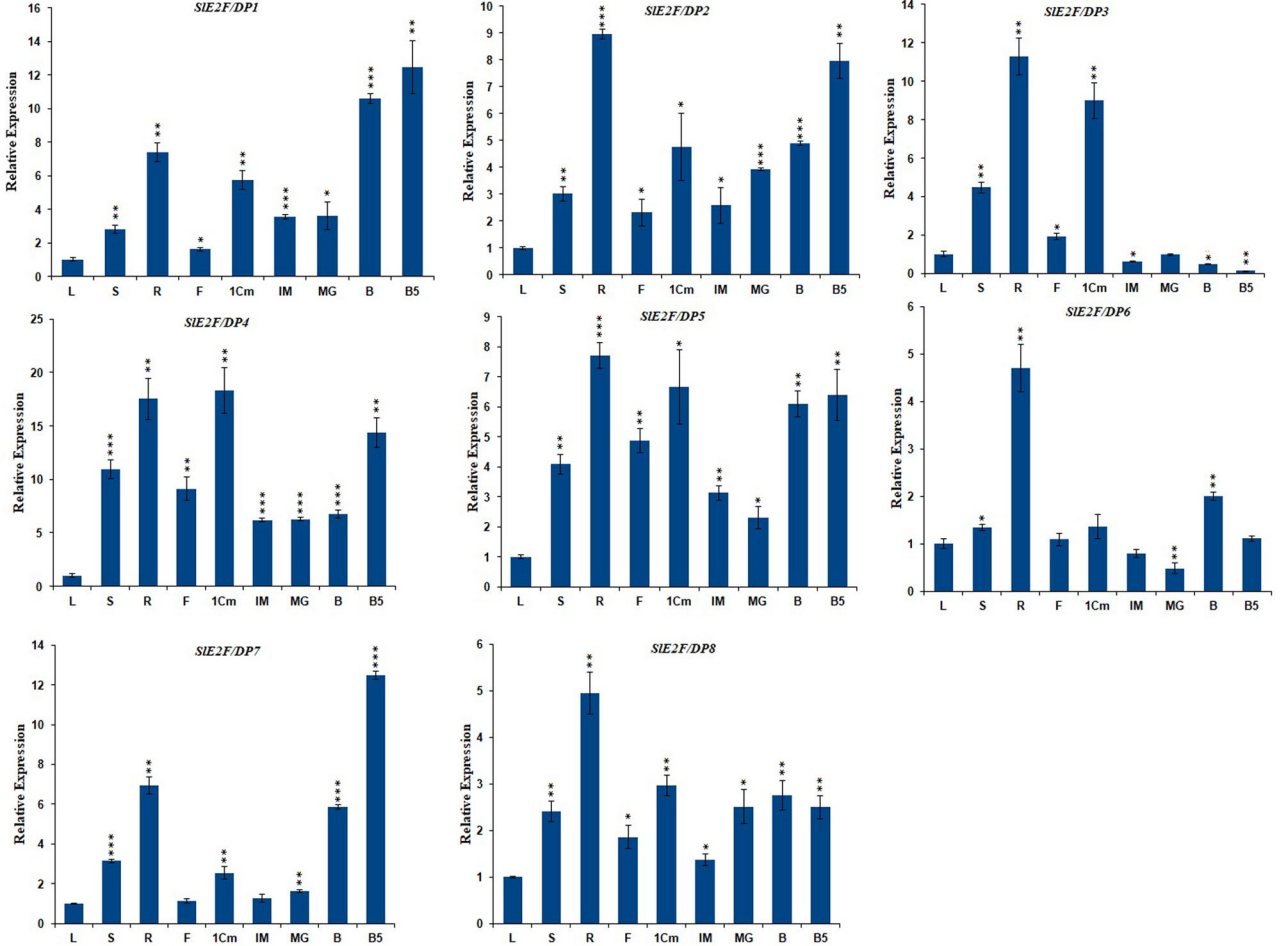
Expression profiles of *SlE2F/DP* genes in various organs: leaves, stems, roots, flowers, 1 cm fruits, immature fruits (IM), mature green fruits (MG), breaker fruits (B), and fruits 5 days after the breaker stage (B5). The standard deviations of the means of three independent biological replicates are represented by the error bars. The different asterisks above the bars indicate the significant variations between the control samples (leaves) and the other organs by Student’s t-test with p‐values less than 0.05 for *, 0.01 for **, and 0.001 for ***, respectively

Further to understand the functional role of these *SlE2F/DP* genes in different developing tomato organs, expression analysis was conducted (roots, stems, leaves, flowers, fruits: 1 cm, IM, MG, B, and B5) of the Alisa Craig genotype through RT-qPCR. (Fig. 8). The expression levels of eight *SlE2F/DP* genes in tomato organs revealed differential expression patterns (Fig. 8). The duplicated gene pair, *SlE2F/DP*1 and *SlE2F/DP*5, showed high expression levels in roots (> 4-fold) compared to the control (leaf) (Fig. 8). Similarly, another duplicated gene pair *SlE2F/DP*2 and *SlE2F/DP*3, also showed higher expression in roots (> 8‐fold) than the control. The *SlE2F/DP*1 gene, showed higher relative expression in B5 (12 fold change) followed by B fruit (10 fold), roots (7 fold change), 1 cm fruits (5 fold change), MG fruit (3 fold change), IM fruit (3 fold change), stem (2 fold change), but the expression in flowers was lower than that in the control (Fig. 8). Three genes *SlE2F/DP*2, *SlE2F/DP*4 and *SlE2F/DP*5 were significantly upregulated in all the samples (Fig. 8). *SlE2F/DP*2 had higher expression in the root (9 fold) and B5 (8 fold), followed by 1 cm fruit (5 fold) and B (4 fold), stem and MG (3 fold) and flower and IM (2 fold) compared to its respective control (Fig. 8). *SlE2F/DP*4 gene expressed highly in 1 cm fruit, root, B5, stem, flower, B, MG, IM by 18, 17,14,10,9, 6.7, 6.2 and 6.2 fold values (Fig. 8). Similarly, *SlE2F/DP*5 gene had high expression in root (7.7 fold) followed by 1 cm fruit, B5, B, flower, stem, IM and MG (6.6, 6.4, 6.0,4.8, 4,3.1 and 2.3 fold) (Fig. 8). The gene *SlE2F/DP*3 showed high expression in root sample (11 fold) followed by 1 cm fruit (8 fold), stem (4 fold) compared with their control. However, its expression level was less than the control in other samples. Similarly, *SlE2F/DP*6 expressed more than 2-fold in the root (4.7) and B fruit (2.0), but the expression was less than the control in other samples. *SlE2F/DP*7 showed high relative expression in B5, root, B, stem and 1 cm fruit (12, 6.9, 5.8, 3.1 and 2.5) and the remaining samples were less than 1 fold. *SlE2F/DP*8 gene showed 4.9 fold in the root, and in five samples (stem, 1 cm, MG, B, B5) more than 2 fold change than the control sample.

### Expression analysis of *SlE2F/DP* genes against abiotic stresses and phytohormone treatment

**Fig. 9:**
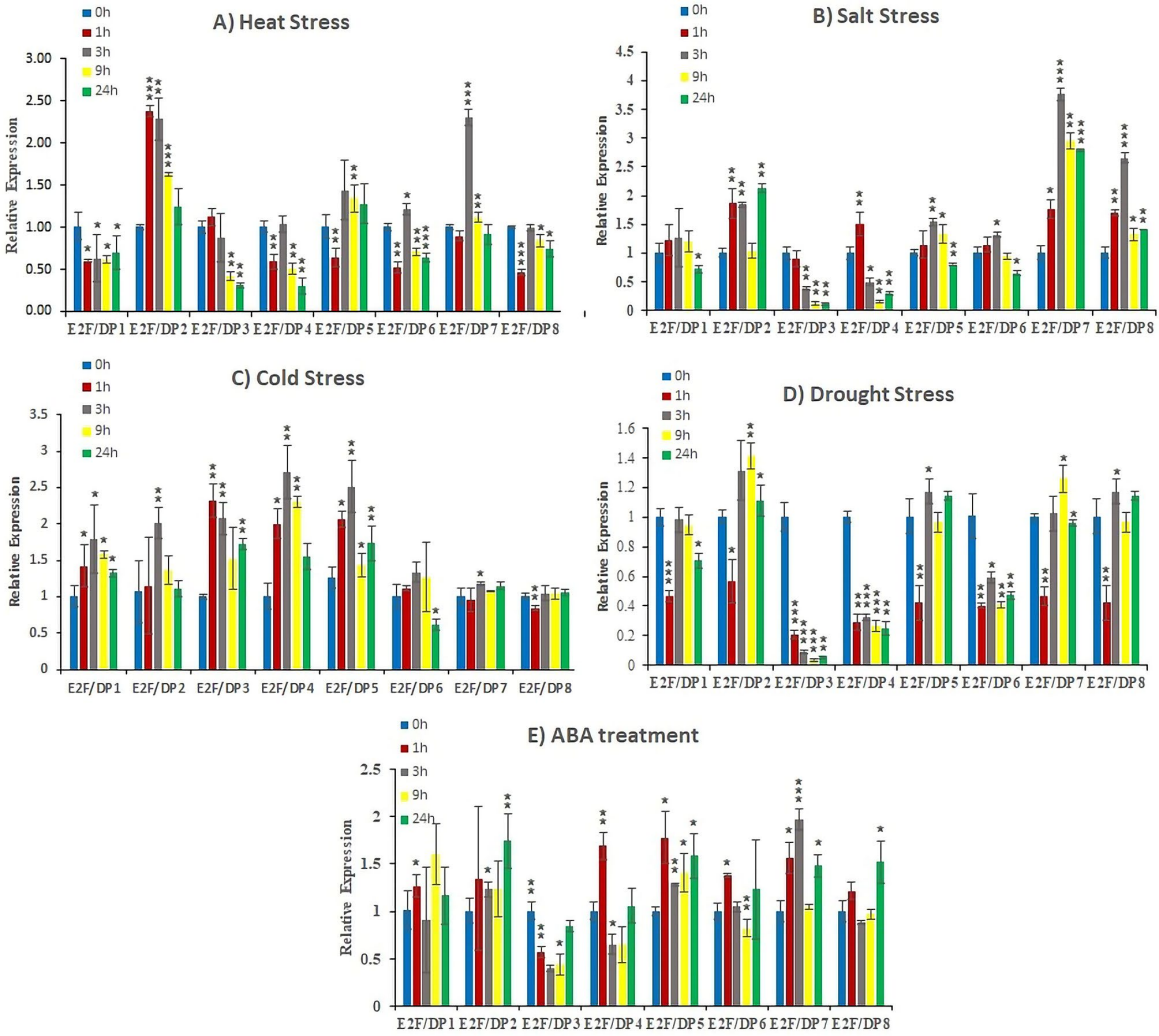
Expression profiling of *SlE2F/DP* genes under different abiotic stresses: (**a**) heat, (**B**) salt, (**C**) cold, (**D**) drought, and (**E**) ABA treatment. Error bars represent standard deviations of the means of three independent biological replicates. The various asterisk marks (* for p-value < 0.05, ** for p‐value < 0.01, and *** for p‐value < 0.001) indicate statistically significant compared to its respective control using the student t‐test

The transcript profiles of tomato *E2F/DP* genes were studied in leaf samples exposed to different abiotic stresses (heat, drought, cold, and salt) and phytohormone (ABA) treatments through qRT-PCR (Fig. 9A–E). Six genes (*SlE2F/DP*1, *SlE2F/DP*3, *SlE2F/DP*4, *SlE2F/DP*5, *SlE2F/DP*6 and *SlE2F/DP*8) were significantly downregulated under heat stress at 40 ^o^C (0.30‐ to 1.44‐fold). Interestingly, two genes *SlE2F/DP*2 and *SlE2F/DP*7 were upregulated by 2.30-fold change at 1 h and 3 h under heat stress (Fig. 9A).

Salt stress triggered the expression level of the *SlE2F/DP* genes at various time-points (Fig. 9B). Compared to the control, *SlE2F/DP*2 was upregulated (2.12-fold) at 24 h after salt treatment. Beside *SlE2F/DP*7 gene expression was repressed during the early hours of salt stress but gradually increased after 3 h (3.8 fold) and maintained its expression level until 24 h (Fig. 9B). In contrast, *SlE2F/DP*8 gene was upregulated by 2.7 fold change at 3 h and gradually decreased at later stages after salt stress.

Seven *SlE2F/DP* genes were significantly upregulated at several time points against the cold stress (4 ^o^C). *SlE2F/DP*2 gene showed significant upregulation of 2-fold change at 3 h of exposure to cold stress. Similarly, *SlE2F/DP*3 and *SlE2F/DP*5 genes showed upregulation (> 2 fold) at 1 h and 3 h against the cold stress. In contrast, *SlE2F/DP*4 gene showed > 2 fold upregulation at the early stages and gradually decreased at 24 h under cold stress. Remaining four genes *SlE2F/DP*1, *SlE2F/DP*6, *SlE2F/DP*7 and *SlE2F/DP*8 were downregulated (0.61 to 1.79 fold) compared to the control (Fig. 9C).

Drought stress-regulated the expression levels of the *SLE2F/DP* genes. Four genes *SlE2F/DP*1, *SlE2F/DP*3, *SlE2F/DP*4 and *SlE2F/DP*6 were significantly downregulated under drought stress. Another four genes *SlE2F/DP*2, *SlE2F/DP*5, *SlE2F/DP*7 and *SlE2F/DP*8 were upregulated by 1.1 to 1.4 fold under drought treatment compared with the respective control (Fig. 9D).

The *SlE2F/DP* genes expression level was similar to control after the ABA hormone treatment. Only *SlE2F/DP*7 gene showed 2-fold upregulation at 3 h compared to the control in response to ABA treatment. Six genes (*SlE2F/DP*1, *SlE2F/DP*2, *SlE2F/DP*4, *SlE2F/DP*5, *SlE2F/DP*6 and *SlE2F/DP*8) were expressed from 1.1 to 1.8 fold at various time points under ABA treatment compared with the respective control. In contrast, *SlE2F/DP*3 gene was downregulated at all the five time points tested (Fig. 9E).

## Discussion

*E2F/DP* transcription factors (TFs) have been identified in both plants and mammals. The classification of plant *E2F/DP* genes is generally based on their phylogenetic analysis, domains, motifs, and subcellular localization, and their role against abiotic stress has been reported in few plant species [19, 20, 32], but not in any solanaceous crop. The present study identified and characterized eight *E2F/DP* genes of tomato. The number of *E2F/DP* genes was observed to vary from one species to another within the plant kingdom. Hence, these genes may play an important biological role in this vegetable crop.

Based on the phylogenetic analysis the E2F/DP homologs mostly from the dicot species, and the monocot species, clustered separately suggested that these proteins may be separated during the divergence of monocots and dicots (Fig. 1). However, in group II, only the tomato and potato proteins were clustered, revealing that these proteins were particular to solanum families among the dicots. In contrast, group IV clustered both the monocot and dicot plant species, suggested that they originated from the common ancestor angiosperm before the divergence of monocot and dicot (∼200 million years ago) [33]. Notably, all the SlE2F/DP proteins formed a close association with potatoes E2F/DP proteins, underlying the evolutionary conservation of these *E2F/DP* members in the solanaceous family.

The conserved domain analysis results suggested that all the SlE2F/DP proteins had E2F-TDP domain along with or without additional domains (Fig. 2). These findings suggested that the combination of additional domains may have evolved and differed from the ancestral *E2F/DP* domains. All these *SlE2F/DP* genes were analysed for the conserved motifs and gene structure prediction suggested that structural differences may be particular to each gene family (Fig. S1, Fig. S2). The similar motifs of paralogous and orthologous gene pairs from tomato, rice and *Arabidopsis* suggested that these conserved motifs remain similar even after species evolution between monocots and dicots of SlE2F/DP family genes.

During the evolutionary process, gene duplication events are the primary sources of genetic novelty [34]. This segmental duplication increases the number of genes within a particular gene family, which enhances plants adaptation to various environmental stresses [35, 36]. The two duplication pairs in the SlE2F/DP gene family suggested that segmental gene duplication triggered the expansion of the tomato E2F/DP gene family during evolution. Multiple segmental duplication events were reported in the E2F/DP gene family in wheat [20]. Another study reported that 12 duplicated gene pairs were observed in the E2F/DP gene family in Moso bamboo [32]. In contrast, no gene duplication was observed in the E2F/DP gene family in *Medicago truncatula* [19].

Microsynteny analysis revealed six segmental gene pairs between tomato and *Arabidopsis* and divergence time was calculated for dicot (Table 2) indicate that tomato has the closest relationship with the dicotyledonous model plant *Arabidopsis*, than the monocotyledonous model plant rice (Fig. 4). Interestingly, the three tomato genes were commonly duplicated between tomato and *Arabidopsis* as well as between tomato and rice. The duplication of gene pairs observed between *Arabidopsis* and rice suggested that these genes could have been present in *Arabidopsis* and rice before the separation of ancestral genes during the evolutionary process. Moreover, three tomato genes were commonly duplicated between the dicotyledonous plant *Arabidopsis* and the monocot rice plant suggested that these genes play an important role in evolutionary changes (Fig. 4).

The cis-regulatory elements in the promoter region has been reported to be associated with stress-related genes against biotic and abiotic stress [37, 38]. In the present study, we also predicted numerous hormone-related and abiotic stress-responsive *cis-*acting elements in the promoter regions of 8 *SlE2F/DP* genes (Fig. S6 and Table S2). Low-temperature responsive element (LTR) motifs were present in the promoter regions of *SlE2F/DP*1 and *SlE2F/DP*4. All the 8 *SlE2F/DP* promoter regions had more than two light response (TCT, GT1, GATA, GA, G-box, I-box and ACE) motifs. Similarly, all 23 *PheE2F/DP* genes had light response motifs in their promoter region [32]. The drought-responsive element MBS motif was detected in the promoters of *SlE2F/DP*6, *SlE2F/DP*7 and *SlE2F/DP*8 suggested their role in abiotic stress. These MBS elements were reported in *HVA22* gene and *PLATZ* transcription factor against abiotic stress [39, 40]. The ABRE cis-elements and the Meja responsive motif is abundant in *SlE2F/DP* genes. In addition, auxin, salicylic acid and gibberellin-responsive elements were also observed in some of the *SlE2F/DP* genes, suggested that they may be stimulated by environmental stress. This type of hormone cross-talk regulatory element was also reported in *E2F/DP* genes of Moso Bamboo [32].

miRNAs can regulate gene expression either by inhibiting transcription and translation or by suppressing transcription and translation [41]. In the current study, six tomato miRNAs (sly-miR172a, sly-miR172b, sly-miR9476-5p, sly-miR9472-3p, sly-miR9471a-3p and sly-miR9471b-3p) were targeted to cleave four *SlE2F/DP* genes (Table S3). Sly-miR172a and Sly-miR172b were reported to be involved in tomato fruit development, ripening and against salt and drought stress [42, 43]. Similarly, other miRNAs, namely, Sly-miR9476 and Sly-miR9472 were reported to be involved in the regulation of defence genes *DRP*, *HD-ZIP*, *MYB*, and *NAC* against drought stress [44]. Another sly-miR9472 was reported to be involved in drought-stress through RNA sequencing and qRT-PCR [45]. Interestingly, *SlE2F/DP*1 and *SlE2F/DP*8 were predicted to have targets for multiple miRNAs. These results suggested that multiple miRNAs could regulate the gene expression of *SlE2F/DP* genes in tomatoes.

Protein phosphorylation plays an important role in post-translational modification by altering the protein structure or its function. To activate or deactivate the proteins, the amino acids are phosphorylated by kinases and phosphatases that are involved in signalling pathways and stress responses in plants [46, 47]. This study predicted phosphorylation sites in all tomato E2F/DP proteins (Table S4). Protein kinases are reported against cold and heat stress in tomato, *Arabidopsis*, wheat, and soybean [48, 49]. The phosphorylation sites S686, T688, S689, and T705 act as targets for mutagenesis and are required to maintain Xa21 protein stability of rice bacterial blight disease [50]. Similarly, phosphorylation sites T867, S878, T1040, and T1072 is also essential for the function of the *FLS2* gene against bacterial *Pseudomonas syringae* in *Arabidopsis* [51]. In the recent years, around 1098 phosphorylation sites were reported in response to drought or heat stress in the grass species [52]. However, the exact role of these phosphorylation sites can be elucidated after conducting additional experimental studies.

To understand the possible interaction with other molecules and ligand-binding sites, a three-dimensional structure of SlE2F/DP proteins was predicted (Fig. 5). Most of the E2F/DP proteins had a higher number of coils than α-helixes and β‐strands (Table S5) suggested its important role in the molecular structure during evolution. Although it has yet to be experimentally validated, the SlE2F/DP proteins were predicted to have a 3D modelling structure with their binding molecules such as ligand binding sites, ions, including peptide molecules and DNA that can change cell function.

The transcription factors are reported to be localized in the nucleus. The subcellular localization results showed that only SlE2F/DP3 localized in the nucleus and three proteins SlE2F/DP4, SlE2F/DP6 and SlE2F/DP7, were predominantly localized in the ER (Fig. 7). Even though, TFs are reported to be localized in the nucleus, some TFs (bZIP and NAC) families are not localized in the nucleus during their initial synthesis [53]. In the genome wide characterization of MYB TFs in *Physcomitrella paten*, one gene was localized in the ER [52]. Similarly, the CREB3 (bZIP) family of TFs are ER-localized proteins [54]. In contrast, to our results E2F/DP proteins were expressed in the nucleus and cytoplasm in wheat [20]. These results suggested that SlE2F/DP3 may act as a transcription repressor and that other E2F/DP proteins may act as co-activators which is required for E2F activity or in regulating signalling pathways [55].

Gene expression studies of SlE2F/DP family genes were tested in various tissues to understand their functional role and diversity in tomatoes. Interestingly, many of the E2F/DP family genes namely *SlE2F/DP*3, *SlE2F/DP*6 and *SlE2F/DP*8 were predominately expressed in the vegetative tissues (roots), while other genes, *SlE2F/DP*1 and *SlE2F/DP*7 were highly expressed in the reproductive tissues (B5), suggested its importance in the tissue-specific functions and development in tomato. The remaining three genes *SlE2F/DP*2, *SlE2F/DP*4 and *SlE2F/DP*5 showed upregulation in both vegetative and reproductive tissues. All the eight tomato *E2F/DP* genes that were highly expressed in roots and stems, suggested their involvement in root growth is to uptake and supply nutrition and water to other parts of plants. A similar trend was observed in most of the *E2F/DP* gene expression studies in *Medicago truncatula* [19]. In contrast, the most of the *E2F/DP* genes are highly expressed in leaf tissues in wheat [20].

The role of *E2F/DP* genes in fruit development has not been studied in any vegetable crop, therefore our study is the first analysis of *E2F/DP* genes in the reproductive stages of tomato. Out of the eight *E2F/DP* genes, four genes namely *SlE2F/DP1*, *SlE2F/DP2*, *SlE2F/DP4* and *SlE2F/DP*5 were expressed in both 1-cm and breaker stages suggested that these genes may be involved in both cell division and ripening processes. Another three genes namely *SlE2F/DP*1, *SlE2F/DP*2 and *SlE2F/DP*7 were highly expressed in the B5 followed by the B revealing that these genes may play an important role in the fruit ripening processes. The higher expression levels (≥ 5 fold change) of *SlE2F/DP*1, *SlE2F/DP*3, *SlE2F/DP*4 and *SlE2F/DP*5 in 1-cm fruit suggested that they may be involved in the cell division phase of tomato fruit development. In another study, *SlE2F/DP*3 (*Solyc03g113760*) was reported to be involved in tomato fruit development [56]. In contrast, *SlE2F/DP*6 gene showed lower expression levels in all developmental stages of fruit. These results suggested that *E2F/DP* genes play a regulatory role in fruit development, enlargement and ripening, which may lay the foundation for functional validation of this gene family in tomatoes.

Plants have been exposed to many wide ranges of environmental stress and biotic stress. Plant defence against abiotic stress was regulated by a complex network of genes including numerous transcription factors, defence genes along with its downstream genes reported to be involved in the ABA-mediated pathway [57]. A few reports of *E2F/DP* transcription factors in plant abiotic stress have been reported in different plant species [19, 20, 32].

In the current study, tomato *SlE2F/DP* genes displayed different expression levels against different abiotic stress (Fig. 9A-E). Out of the eight genes tested, two genes showed significant upregulation against heat stress (Fig. 9A), which agrees with the earlier finding of *E2F/DP* genes in wheat in response to heat stress [20]. Salt stress triggered the expression level in three genes *SlE2F/DP*2, *SlE2F/DP*7 and *SlE2F/DP*8 (Fig. 9B). These results suggested their possible role in salt tolerance in tomato. Similarly, most of the *E2F/DP* genes were expressed significantly in Moso bamboo and barrel clover upon exposure to salt stress [19, 32]. In contrast, the *E2F/DP* genes were expressed less than two-fold at different time points in wheat [20]. The expression level of four *SlE2F/DP* genes (*SlE2F/DP*2, *SlE2F/DP*3, *SlE2F/DP*4 and *SlE2F/DP*5) were induced following cold stress (Fig. 9C). These results were consistent with previous reports of three wheat *E2F/DP* genes (*TaE2F11-9*, *TaDP3111-15* and *TaDEL211-27*) in response to cold stress [20]. On exposure to drought stress, the expression level of *SlE2F/DP* genes was induced ≤ 1.2 fold at any time point. Our results were consistent with a previous report of *E2F/DP* genes in wheat [20]. The phytohormone ABA is well-studied and known for its role in regulating many stress related genes against various environmental stresses [58, 59]. Our results showed that *SlE2F/DP*7 was upregulated only at one-time point in tomato plants following ABA treatment (Fig. 9E) suggested its possible role in drought tolerance. To understand the defence mechanism against rice blast infection, 14 different TFs were used for that study. Interestingly, the *E2F/DP* TF gene of rice (LOC_Os12g06200.1) was upregulated at the early stage against the blast infection [60]. Till now, no reports of *E2F/DP* genes treated with ABA phytohormones in other plant species. Besides, key cis-regulatory elements related to hormones-responsive, including ABA, auxin, SA, and jasmonic acid were observed in upstream site of *SlE2F/DP* genes. It was stated that phytohormones such as ABA, SA, and jasmonic acid are associated with plant cell signalling pathways linked to response of biotic and abiotic stresses [61]. Key stress signalling pathways and ion transporter genes are controlled by ABA signalling in response to stresses [62]. Our results disclose that *SlE2F/DP* genes are probably being in cellular cascades related to ABA.

To gain a better understanding of the putative functions of *SlE2F/DP* genes, gene co-expression network analysis of *SlE2F/DP* was performed using the RNA-seq data (Fig. 6). Several genes involved in the co-expression network interaction were related to abiotic stress tolerance. *SlE2F/DP3* co-expressed genes such as *SlHSP SlLRR, SlF-box-LRR, NAC* domain gene and B3 DNA binding domain were reported against biotic and abiotic stress [19, 34, 63, 64]. Similarly, another gene thymidylate synthase reported to control the expression of folate biosynthesis genes in tomato fruit was co-expressed with the *SLE2F/DP*5 gene [65]. In addition, another *SlHSP20* involved in heat tolerance in tomato and *SlGDSL* gene regulates fruit development in tomato [66, 67] were detected in the co-expression analysis of the *SlE2F/DP*7. Likely, *SlE2F/DP*8 gene established a co-expression network with several genes such as Zinc finger C3HC4, CCCH domain, serine/threonine and cyclin-related gene are reported to be involved in both biotic and abiotic stress and cell division in tomato [68–70]. These results suggested that these transcription factor *E2F/DP* genes may play an important role in abiotic stress tolerance in tomato.

## Conclusion

In the present study, we identified eight *SlE2F/DP* genes in tomato. These genes were clustered into seven phylogenetic groups based on their domain, gene structure and motif analyses. Gene duplication analyses spotted the two segmental gene duplication events were responsible for the expansion of the E2F/DP gene family throughout the evolution in tomato. We hypothesize that tomato *E2F/DP* genes play an important role in development process. In addition, *E2F/DP* genes were associated with cis-regulatory elements, miRNA target sites and phosphorylation sites in their sequences in regulating gene expression. Expression profiling showed that most of the *SlE2F/DP* genes are highly expressed in both vegetative and reproductive organs. Many of the *SlE2F/DP* genes were significantly induced under different abiotic stress concluding that their possible function in tolerance against this abiotic stress. Co-expression network analysis highlighted the biological importance of *SlE2F/DP* genes in tomato and predicted the hub genes co-expressed with *SlE2F/DP* genes revealing their possible role in development and abiotic stress tolerance. Our findings provide the valuable information for the further functional exploration of the SlE2F/DP gene family and identify the potential candidate genes for the genetic improvement of tomato.

## Methods

### Identification and sequence analysis of *E2FDP* genes in the tomato genome

We identified the *E2F/DP* genes in the Plant Transcription Factor Database (http://www.Planttfdb.gao-lab.org) using the keyword search “*E2F/DP*’’ [71]. Simultaneously, E2F/DP protein sequences were downloaded from the TAIR database (https://www.Arabidopsis.org) of *Arabidopsis thaliana.* We performed a blast search using AtE2F/DP protein sequences in the Sol genomics database (http://www.solgenomics.net/tools/blast) with its default parameters [72]. The HMM profile of SlE2F/DP1 (PF16421) was taken from the Pfam (http://pfam.xfam.org/). The eight SlE2F/DP protein sequences were used for checking the presence of domains using the two online webserver SMART tool (http://smart.emblheidelberg.de/) and NCBI CDD webtool (https://www.ncbi.nlm.nih.gov/Structure/bwrpsb/bwrpsb.cgi). It is essential to observe the molecular weight, protein length, grand average of hydropathicity index (GRAVY), and isoelectric points of the SlE2FDP proteins using the online webserver Expasy (http://cn.expasy.org/tools/protparam.html) [73]. To visualize the exon-intron structure of *SlE2FDP* genes the genomics and coding sequence data were loaded to the Gene Structure Display Server-2.0 (GSDS-2.0) web server (http://gsds.cbi.pku.edu.cn/) [74]. The open reading frames of the *SlE2FDP* genes were analysed using the Open Reading Frame Finder tool (https://www.ncbi.nlm.nih.gov/orffinder/). To identify the conserved domains, full length protein sequences of E2F/DP from *Arabidopsis*, tomato, and rice were analysed using the Multiple EM for Motif Elicitation (MEME) web server (http://meme-suite.org/) with the parameter of 10 motifs with a length of 6 to 50 [75]. Multiple alignment was performed using the protein sequences of *SlE2FDP* genes by the online webtools Clustal Omega and ESPript [76, 77]. The subcellular localization was analysed for SlE2FDP proteins using the webtool “WoLF-PSORT” (https://wolfpsort.hgc.jp/) [78]. The sequence homology of SlE2FDP proteins was analysed using the webserver “Immunomedicine Group” (http://imed.med.ucm.es/Tools/sias.html).

### Phylogenetic analysis of tomato E2F/DP proteins

The E2F/DP protein sequences were downloaded from the nine different plant species and aligned using the online server ClustalW and then the aligned file was used for phylogenetic analysis using the method the neighbour-joining (NJ) with the 1000 bootstrap replications in MEGA 6.0 software [79]. The nine plant species sequences of *Arabidopsis thaliana*, *Brachypodium distachyon*, *Glycine max*, *Oryza sativa*, *Populus trichocarpa*, *Solanum lycopersicum*, *Solanum tuberosum*, *Triticum aestivum*, and *Zea mays* sequences were taken from the Phytozome database http://www.phytozome.net. All the sequences, gene names and accession number details used in this study are given in Table S13.

### Chromosomal locations, gene duplication, and microsynteny analysis

For all the eight *SlE2F/DP* genes the chromosomal locations were collected from the Sol genomic database (http://www.solgenomics.net). With the gene physical position and chromosome details these genes were mapped on their respective chromosomes using the online server MapGene2Chrom web v2 (http://mg2c.iask.in/mg2c_v2.0/). The duplicated gene pairs among the eight *SlE2F/DP* genes were predicted using the one-step MCScanX program of TBtools software [80] and followed by BLASTP program with an E-value < 10 − 10. The duplicated gene pairs, synonymous (Ks) and non-synonymous (Ka) ratio were calculated with the Nei and Gojobori (NG) program of TB tool software [81]. Furthermore, the mode of selection was calculated based on the Ka/Ks ratio [80]. This duplicated gene pair’s divergence time (T) was calculated with the standard formula T = Ks/2r MYA (millions of years ago). Ks-stands for synonymous substitution rate per site and r stands for 1.5 × 10 − 8 substitutions per site/year for all the dicot species [81]. The *E2F/DP* genes from the tomato, *Arabidopsis*, and rice was performed for the synteny analysis using the reciprocal BLAST search program of TB tool software. The duplicated gene pairs across the three genomes were visualized using the circos program of the TB tool software [80].

### Prediction of cis-acting elements, mirna target sites, phosphorylation sites

The putative miRNA present in the coding region of *E2F/DP* genes was predicted using the online tool psRNATarget (http://plantgrn.noble.org/psRNATarget/analysis). Likewise, the cis-acting elements existing in the promoter (1500 bp) of *SlE2F/DP* genes were analysed using the online webserver Plant-Care database (http://bioinformatics.psb.ugent.be/webtools/plantcare/html/) [82]. The phosphorylation sites (Ser/Thr/Tyr) was predicted in the *SlE2F/DP* genes using the online web-based tool NetPhos 3.1 [83].

### Homology modelling of SlE2F/DP proteins

The 3D structure prediction and function of the eight SlE2F/DP proteins was analysed using the web server I-TASSER. These eight SlE2F/DP 3D models were generated using multiple threading alignments with the iterative TASSER and LOMETS assembly programs of the I-TASSER web server [29]. Based on the template, the protein analogs were identified and based on the maximum score and homology in PDB with known structures, the best 3D structures were selected and refined using the ModRefiner program [ 84]. The 3D structure of SlE2F/DP proteins along with their ligand-binding sites were visualized using Discovery Studio v.21.1 software.

### Plant sample collection and stress treatments

Tomato seeds of the Ailsa Craig genotype (*Solanum lycopersicum L.*) were collected from the Giovannoni laboratory at the Boyce Thompson Institute. The seeds were germinated in the nursery tray and maintained in a growth chamber under the optimum conditions at 25°C day/20°C night, with 16-h light/8-h dark photoperiod, 55–70% humidity, and 300 µmol m^− 2^S^− 1^ of light intensity. Twenty-eight-day old seedlings were used for collecting fresh root, stem, and leaf samples. The remaining plants were moved to the greenhouse under the controlled conditions at 25/20°C day/night temperatures. When the plants reached the reproductive stage the flower and fruit samples were collected for organ specific expression studies. The fruit sample was collected at different development stages (i) after 7 days of pollination with 1 cm diameter (1 cm fruits), (ii) after 21 days of pollination immature fruits (IM fruits), (iii) after 35 days of pollination mature green (MG fruits), (iv) when the fruits reach the breaker stage (B fruits), and (v) after 5 days of breaker stage (B5 fruits) [85].

When the tomato seedlings were 28 days old, the plants were treated with different abiotic stress treatments such as abscisic acid (ABA), cold, drought, heat and salt (NaCl). The leaf samples were collected at five different time points 0, 1, 3, 9, and 24 h after heat at 40˚C, cold at 4˚C, salt (200 mM NaCl) and 100 µM ABA spraying on the leaf samples [37]. In the case of drought treatment, the tomato plants were allowed to withhold water and leaf samples at different time points 0, 24, 48, 60, and 72 h were collected [ 84]. For the control samples (0 h) the tomato seedlings were grown in soil under normal conditions at (25°C) against heat, cold, drought, and ABA stress [ 85]. All the samples were collected for three biological replicates, crushed in liquid nitrogen, and stored at − 80°C.

### Expression profiling of *SlE2FDP* genes by qRT − PCR

Total RNA was isolated from treated and control samples using an RNeasy Mini kit (Qiagen, Germany), as per the manufacturer’s guidelines. The concentration and the purity of RNA samples were checked using NanoDrop® 1000 spectrophotometer (Wilmington, USA). cDNA was synthesized for all the samples with Superscript® III First-Strand kit (Invitrogen, USA) as per the manufacturer’s guidelines. The primers for *SlE2F/DP* genes were designed using Primer3 online software http://frodo.wi.mit.edu/primer3/input.html (Table S12), and each primer pair was validated for specificity using a melting curve analysis [82]. The 18 S rRNA (F: AAAAGGTCGACGCGGGCT, R: CGACAGAAGGGACGAGAC) gene from tomato was used as a normalization gene [86]. qRT-PCR reaction volume of 10 µL contained 1 µL (50 ng) cDNA, 2 µL of 5 pmol concentration primers, 5 µL of iTaq SYBR Green (Qiagen, Hilden, Germany), and 2 µL of double distilled water. The PCR was performed using Quantstudio 5 ® 96 (Applied Biosystems, USA) with following conditions: pre-denaturation at 94 °C for 5 min, followed by 40 cycles at 94 °C for 15 s, annealing at 60 °C for 20 s, and extension at 72 °C for 30 s. The relative expression values were calculated using the 2^−∆∆Ct^ method [87].

### Co-expression network analysis of *SlE2FDP* genes

The RNA-seq data were downloaded from NCBI database (https://www.ncbi.nlm.nih.gov/sra) for the co-expression analysis. The following accessions SRR7652567, SRR7652566, SRR7652565, SRR7652564, SRR7652571, SRR7652570, SRR7652569, SRR7652568, SRR7652563, SRR7652562, SRR7652573, SRR7652572, SRR12026415, SRR12026416, SRR12026417, SRR12026418, SRR12026419, SRR12026420, SRR12026421, SRR12026422, SRR12026423, SRR12026420, SRR12026425, SRR12026426, SRR21847247, SRR21847273, SRR21847264, SRR21847257, SRR21847271, SRR21847272, SRR21847267, SRR21847266 and SRR21847265 were downloaded. The raw sequence quality was checked using the FastQC tool and the low-quality and adapter sequences were removed if any [88]. The good quality reads were mapped on the tomato genome ITAG4.0 using HISAT2 2.1.0 Galaxy platform web server [89, 90]. The reads mapped on the exon were counted using FeatureCounts [91]. The expression of the reads was analysed as fragments per kb per million reads (FPKM) by using DESeq2 software [92]. The co-expression network of *SlE2FDP* genes and WGCNA using the IDEP web tool with genes greater than 1 FPKM [93]. The visualization was constructed using Cytoscape (https://cytoscape.org/). The top ten co-expressed genes were used for GO and KEGG analysis [94], using the KOBAS web tool http://kobas.cbi.pku.edu.cn/.

### Subcellular localization

The coding regions of *SlE2F/DP* genes were amplified with gene-specific primers (Table S11) and then cloned into the sGFP-tagged vector pGA3452 driven by the maize *ubiquitin1* promoter to generate transiently expressed SlE2F/DP–sGFP fusion proteins [95]. A vector expressing NLS-mRFP and OsAsp1-mRFP fusion proteins served as nuclear and ER markers, respectively [96]. The SlE2F/DP–sGFP fusion construct and the *NLS-mRFP* and *OsAsp1-mRFP* constructs were introduced into rice cell protoplasts using PEG-mediated transformation [97]. The samples were incubated overnight at 28°C for a period of 12 to 16 h in the dark condition. After incubation the samples were observed under a confocal microscope (BX61; Olympus, Tokyo, Japan) for GFP and RFP channel fluorescent signals.

### Statistical analysis

The data were analysed using Quantstudio 5 ® 96 inbuild software (Applied Biosystems, USA) with its default baseline and threshold values. Three replicate mean values were tested with Student’s t-tests on MS-Excel 2010 software. Asterisks (*, **, and ***) indicate significance at p-values < 0.05, < 0.01, and < 0.001, respectively.

### Electronic supplementary material

Below is the link to the electronic supplementary material.

Supplementary Material 1

Supplementary Material 2

## Data Availability

We declare that the dataset(s) required to reproduce the results of this article are included in the article and additional file(s) available in the journal webpage.

## Abbreviations

*E2F/DP*: Eukaryotic 2 Transcription Factor/Dimerization Partner
3D: Three Dimensional
GO: Go Ontology
TF: Template Modeling
RMSD: Root-Mean-Square Deviation
C-score: Confident Score
ABA: Abscisic Acid
WGCNA: Weighted Gene Co-expression Network Analysis
