# Supplementary material for: Genome-wide characterization and expression profiling of E2F/DP gene family members in response to abiotic stress in tomato (Solanum lycopersicum L.)
Source: BMC Plant Biol. 2024 May 22;24:436. doi: 10.1186/s12870-024-05107-3 (PMC11110339; doi:10.1186/s12870-024-05107-3)
Supplement: Supplementary file 1 — Supplementary Material 1 [file 12870_2024_5107_MOESM1_ESM.docx]

**Supplementary Materials:** **Genome-wide characterization and expression profiling of *E2F/DP* gene family members in response to abiotic stress in tomato (*Solanum lycopersicum L*.)**

Dhanasekar Divya^1^, Arif Hasan Khan Robin^2^, Lae-Hyeon Cho^3^, Dohyeon Kim^3^, Do-jin Lee^1^, Chang-Kil Kim^4*^, Mi-Young Chung^1*^


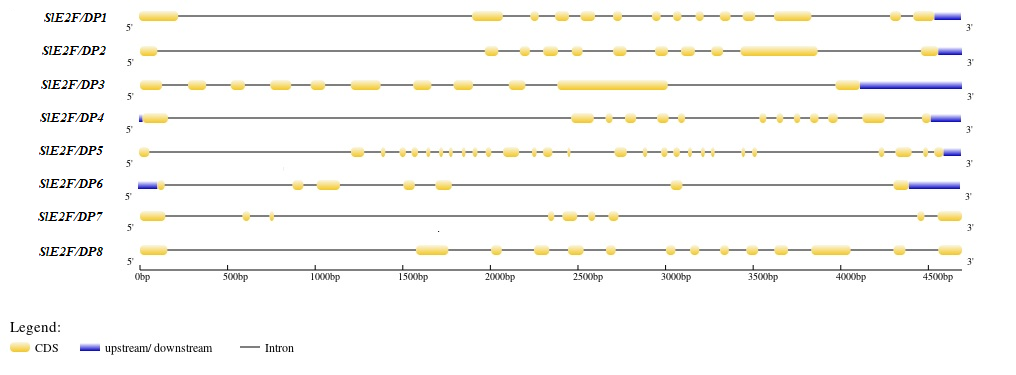


Fig. S1 Schematic depiction of the exon‐intron structure of *SlE2F/DP* genes. Yellow boxes represent exons, black lines indicate introns, and blue boxes denote untranslated regions.


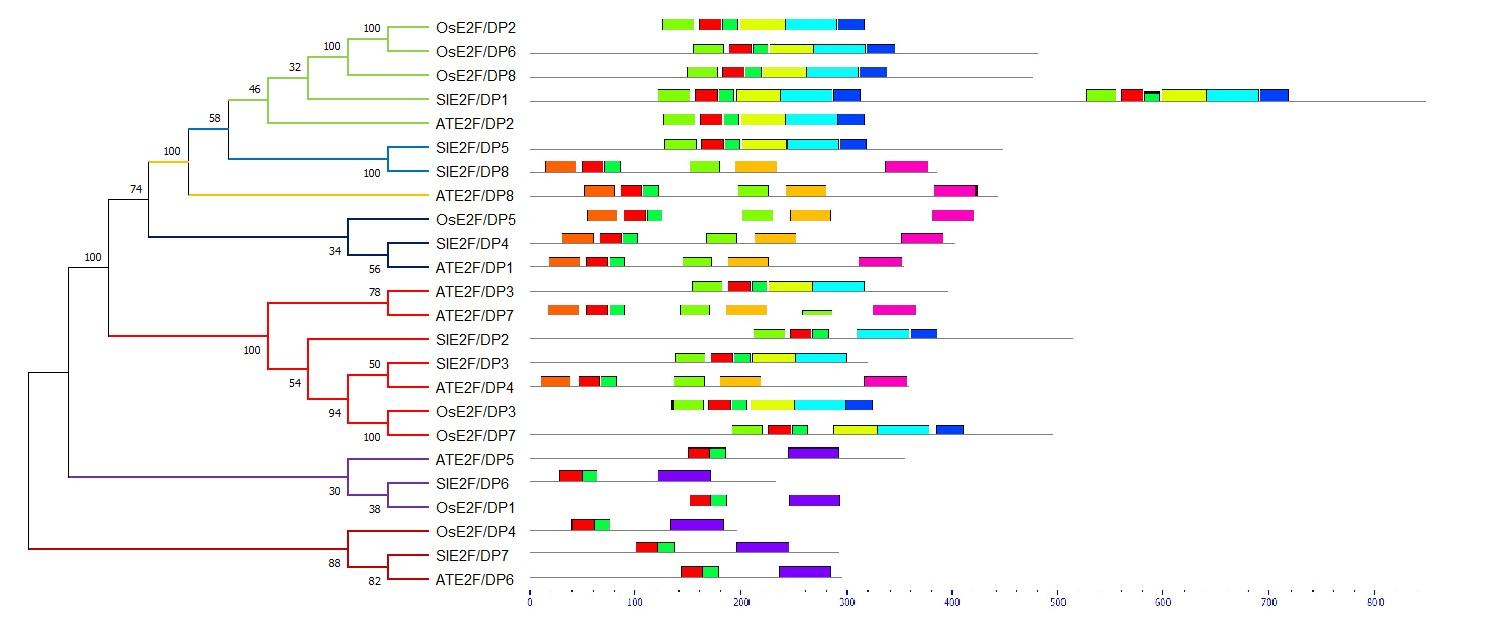


Fig. S2 Conserved motif analysis of E2F/DP proteins from tomato, Arabidopsis and rice. Different colored boxes signify different motifs.


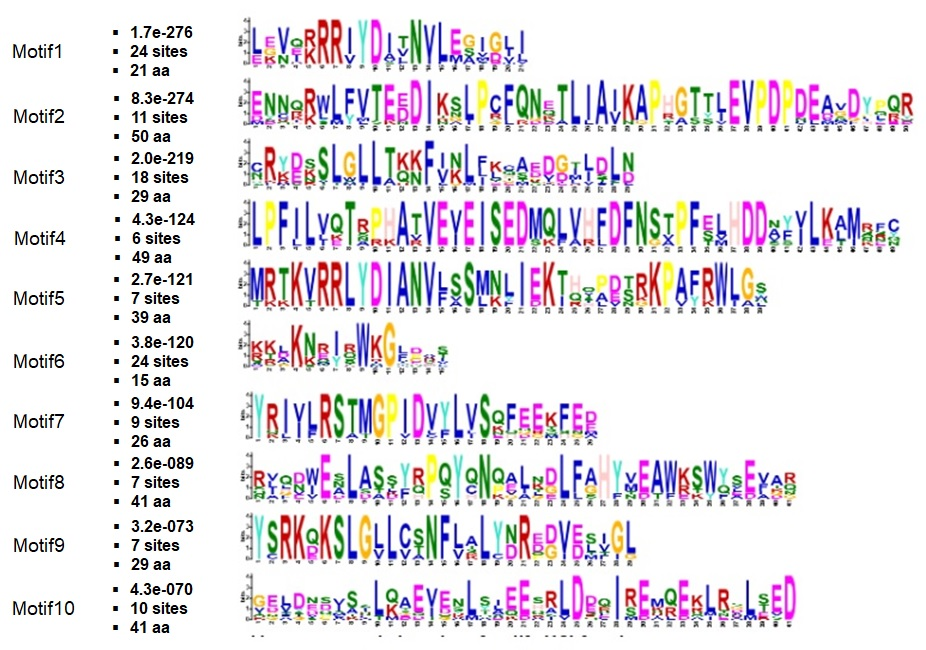


Fig. S3 Overview of conserved motifs of E2F/DP proteins from tomato, Arabidopsis and rice.


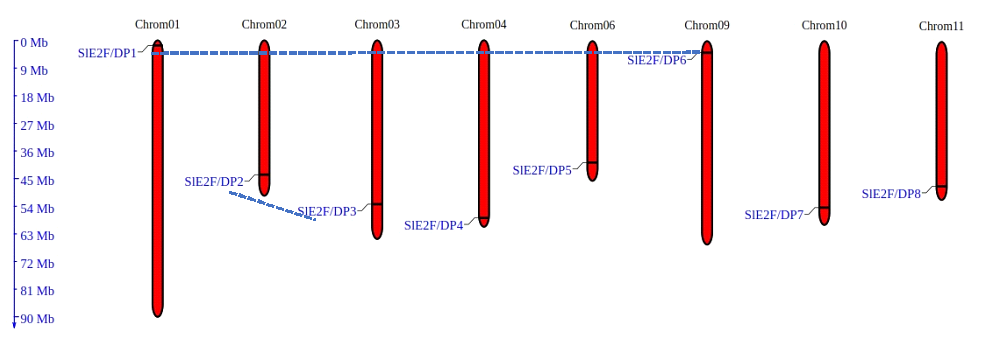


Fig. S4 Chromosome locations of *SlE2F/DP* genes. The chromosome numbers are labelled at the top of chromosomes and the length of chromosomes can be estimated by the scale bar on the left shown in megabases (Mb). The duplicated gene pairs are linked by the blue dotted lines.


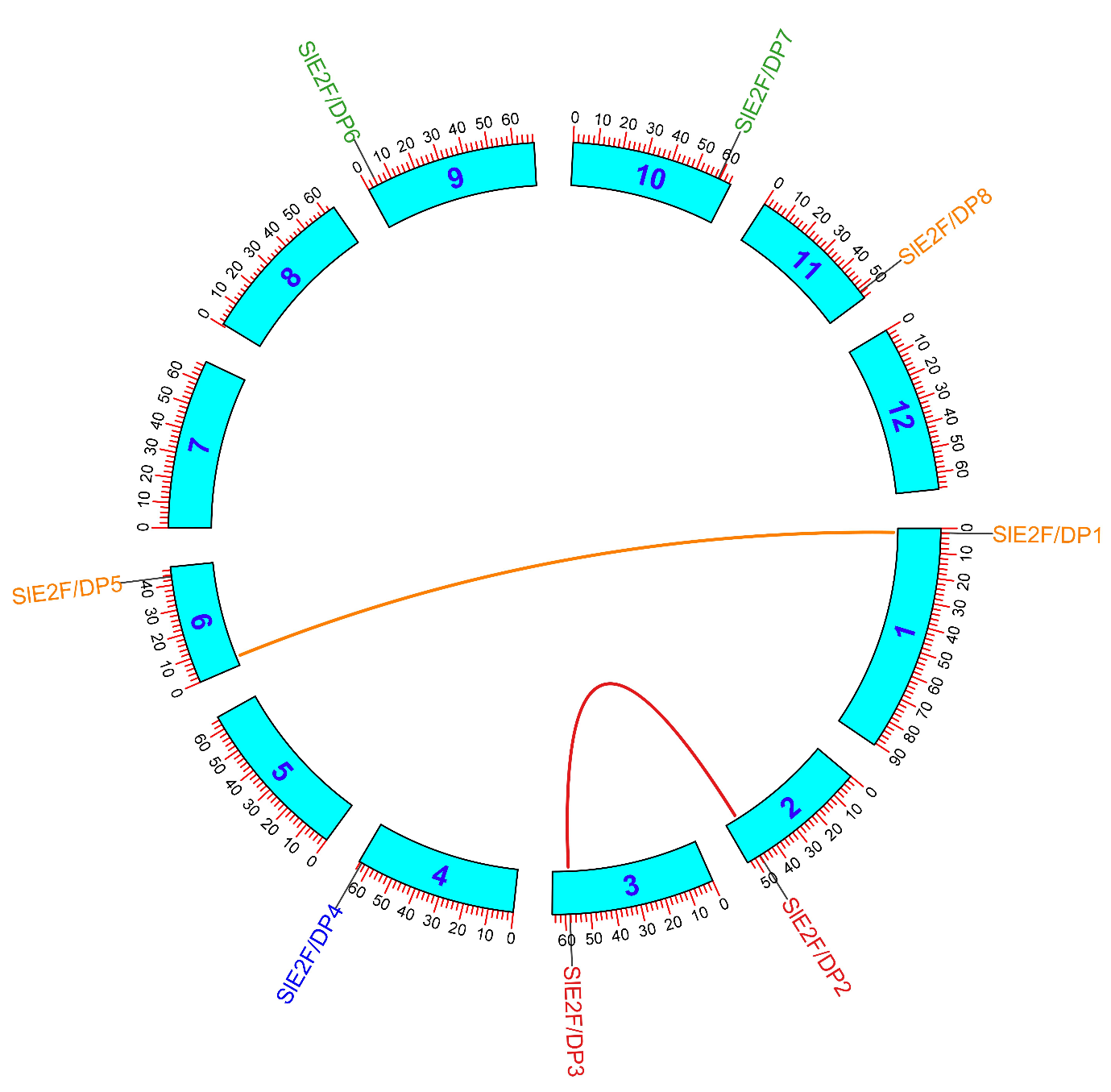


Fig. S5 Gene duplication analysis of *E2F/DP* genes in tomato. Chromosome numbers and gene positions are indicated and the length of each chromosome is displayed on a megabase scale (Mb). The segmentally duplicated gene pairs are interconnected by orange and red lines.


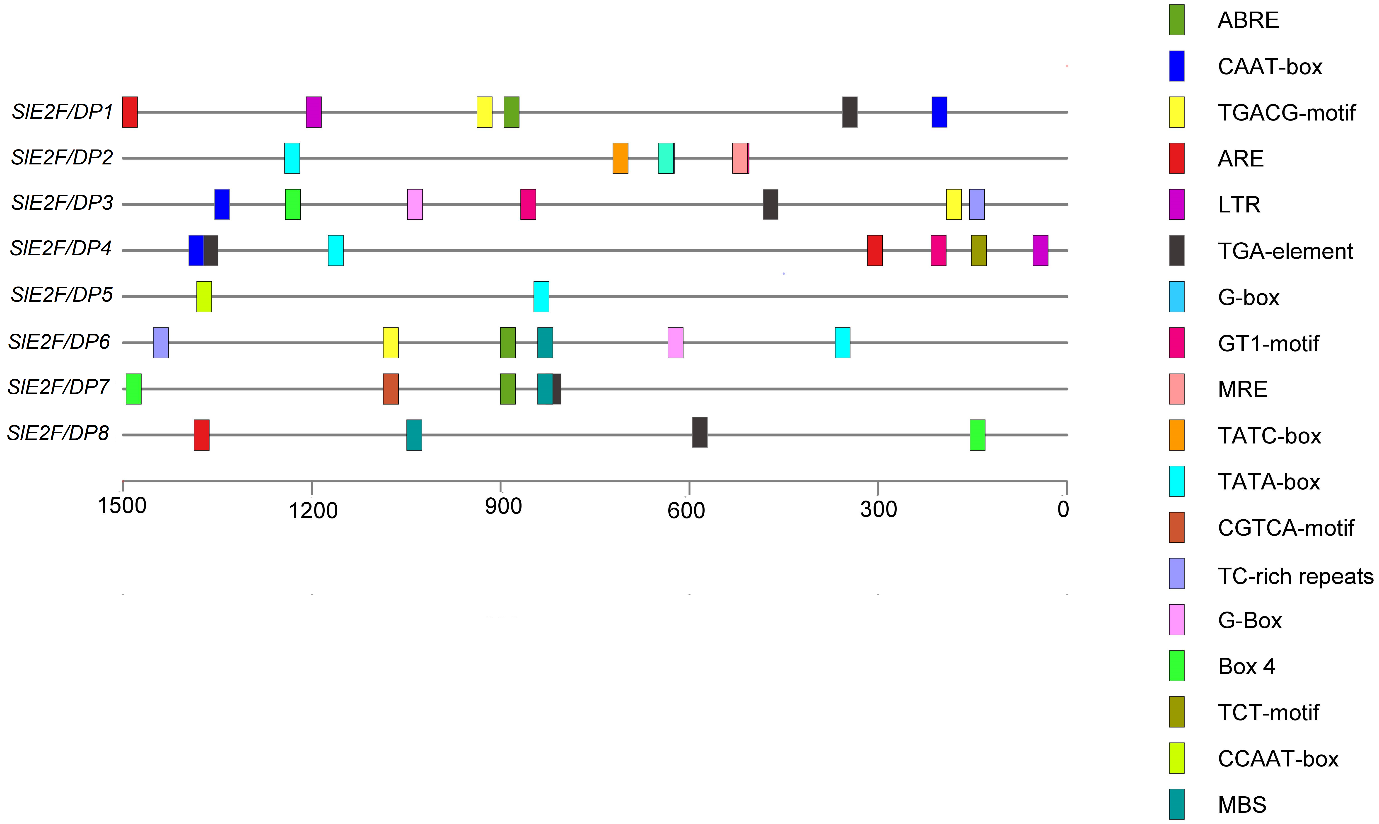


Fig. S6 Predicted cis‐acting elements in the promoters of *SlE2F/DP* genes. The identified elements are: drought‐responsive MYB‐binding site (MBS) and MYB elements, defense and stress‐responsive elements (TC‐rich repeats), jasmonic acid‐responsive elements (CGTCA‐motif), related to the SA response (TCA‐elements), auxin responsive elements (TGA‐element and TGA‐box), ABA‐responsive elements (ABRE), low‐temperature‐responsive elements (LTR), and GA responsive elements (P‐box, GARE‐ and TATC). The scale denotes the positions of cis‐regulatory elements in regard to the translation start site (taken as +1 bp).


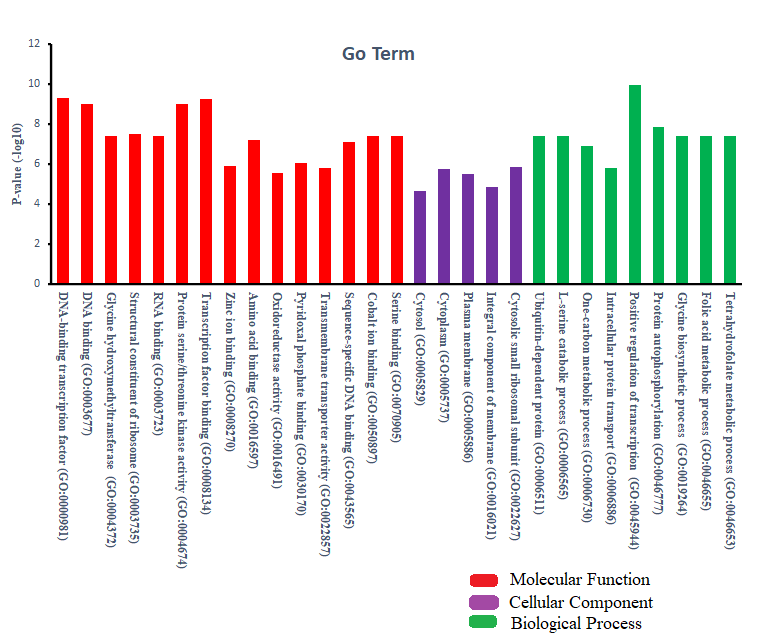


Fig. S7 The enriched GO terms for co‐expressed genes of four *SlE2F/DP* genes.


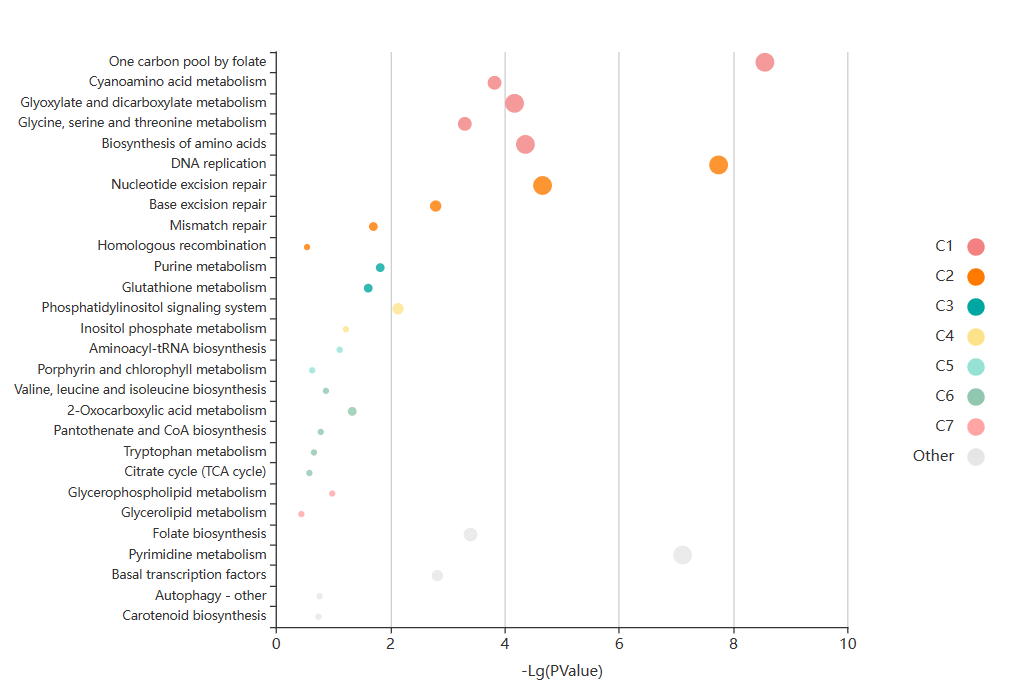


Fig. S8 Bubble chart of the KOBAS enrichment analysis for co-expressed genes with *SlE2F/DP* genes [92]. Each bubble denotes an enriched function, and the sizes of the bubble from small to large reflect the following values: [0.05,1], [0.01,0.05], [0.001,0.01], [0.0001,0.001], [1×10−10,0.0001], [0,1×10−10].
